# Liver RBFOX2 regulates cholesterol homeostasis via *Scarb1* alternative splicing in mice

**DOI:** 10.1038/s42255-022-00681-y

**Published:** 2022-12-19

**Authors:** Helen A. B. Paterson, Sijia Yu, Natalia Artigas, Miguel A. Prado, Nejc Haberman, Yi-Fang Wang, Andrew M. Jobbins, Elena Pahita, Joao Mokochinski, Zoe Hall, Maryse Guerin, Joao A. Paulo, Soon Seng Ng, Francesc Villarroya, Sheikh Tamir Rashid, Wilfried Le Goff, Boris Lenhard, Inês Cebola, Daniel Finley, Steven P. Gygi, Christopher R. Sibley, Santiago Vernia

**Affiliations:** 1 MRC London Institute of Medical Sciences, London, UK; 2 Institute of Clinical Sciences, Imperial College London, Hammersmith Hospital Campus, London, UK; 3 Department of Cell Biology, Harvard Medical School, Boston, MA USA; 4 Division of Systems Medicine, Department of Metabolism, Digestion and Reproduction, Imperial College London, Hammersmith Hospital Campus, London, UK; 5 Sorbonne Université, Inserm, Institute of Cardiometabolism and Nutrition (ICAN), UMR_S1166, Paris, France; 6 Biochemistry and Molecular Biomedicine Department, Institute of Biomedicine, University of Barcelona & Research Institute Sant Joan de Déu, Barcelona, Spain; 7 Centro de Investigación Biomédica en Red de Fisiopatología de la Obesidad y Nutrición (CIBEROBN), ISCIII, Madrid, Spain; 8 Section of Genetics and Genomics, Department of Metabolism, Digestion and Reproduction, Imperial College London, Hammersmith Hospital Campus, London, UK; 9 Institute of Quantitative Biology, Biochemistry and Biotechnology. School of Biological Sciences, University of Edinburgh, Edinburgh, UK; 10 Present Address: Instituto de Investigación Sanitaria del Principado de Asturias, Avenida Hospital Universitario, Oviedo, Spain

**Keywords:** Homeostasis, Metabolism

## Abstract

RNA alternative splicing (AS) expands the regulatory potential of eukaryotic genomes. The mechanisms regulating liver-specific AS profiles and their contribution to liver function are poorly understood. Here, we identify a key role for the splicing factor RNA-binding Fox protein 2 (RBFOX2) in maintaining cholesterol homeostasis in a lipogenic environment in the liver. Using enhanced individual-nucleotide-resolution ultra-violet cross-linking and immunoprecipitation, we identify physiologically relevant targets of RBFOX2 in mouse liver, including the scavenger receptor class B type I (*Scarb1*). RBFOX2 function is decreased in the liver in diet-induced obesity, causing a *Scarb1* isoform switch and alteration of hepatocyte lipid homeostasis. Our findings demonstrate that specific AS programmes actively maintain liver physiology, and underlie the lipotoxic effects of obesogenic diets when dysregulated. Splice-switching oligonucleotides targeting this network alleviate obesity-induced inflammation in the liver and promote an anti-atherogenic lipoprotein profile in the blood, underscoring the potential of isoform-specific RNA therapeutics for treating metabolism-associated diseases.

## Abstract (web summary)

Paterson and Yu et al. demonstrate that loss of the RNA alternative splicing factor RBFOX2 in the liver during a lipogenic diet leads to dysregulation of liver lipid and cholesterol homeostasis through a specific alternative splicing programme, which includes a splice switch of the high-density lipoprotein receptor gene *Scarb1*.

## Main

In mammals, most multi-exon genes undergo AS, generating multiple isoforms^1,2^ and contributing to transcript complexity^3^. AS is thought to be critical for the establishment and maintenance of tissue-specific protein interaction networks^4–6^ and tissue identity^6^. Whether specific AS programs regulate physiological adaptation is less well understood, and their role in metabolic disease is unclear, because of poor functional characterization of specific isoforms and the technical challenges involved in identifying specific upstream regulatory splicing factors in vivo. Thus, there is limited information about splicing networks involved in metabolic reprograming, at the level of both the splicing factors and the isoforms they regulate. As well as increasing our knowledge of metabolic regulation in health and disease, characterization of splicing factors and isoforms involved in metabolic regulation could lead to the development of RNA-based therapeutics to modulate specific isoforms. RNA-based therapeutics to inactivate specific genes in the liver are emerging as promising therapeutic strategies for metabolic pathologies^7,8^. However, splice-switching RNA therapeutics have yet to be explored.

In parallel with the global increase in obesity, metabolism-associated fatty liver disease (MAFLD) has become the most prevalent non-communicable liver pathology^9^. MAFLD ranges from presymptomatic hepatic steatosis (fatty liver) to non-alcoholic steatohepatitis, which is characterized by inflammation, hepatocellular injury and fibrosis, and may progress to liver failure, cirrhosis and hepatocellular carcinoma (HCC)^10^.

While the exact mechanisms promoting the progression of steatosis to non-alcoholic steatohepatitis are not fully understood, evidence suggests that chronic overnutrition and hypercaloric western-style diets contribute to the dysregulation of bioactive and/or toxic lipid species, such as phospholipids, saturated fatty acids, sphingomyelins, ceramides and cholesterol^11–13^. In addition, there is evidence that MAFLD contributes to the pathogenesis of type 2 diabetes and cardiovascular disease, with coronary artery disease being the principal cause of death in these patients^14,15^. Understanding how MAFLD is linked with increased cardiovascular risk and progressive liver damage will aid in identifying new therapeutic targets.

Here, we find that components of the pre-mRNA AS machinery are selectively regulated by metabolic inputs in the liver. We identify RNA-binding fox-1 homolog 2 (RBFOX2) as a key splicing factor in the liver, regulating AS in a cluster of genes involved in lipid homeostasis, including scavenger receptor class B type I (*Scarb1*), phospholipase A2 group VI (*Pla2g6*), the clathrin vesicle adapter *Numb*, a component of the COPII vesicle trafficking system *Sec31a* and oxysterol-binding protein-like 9 (*Osbpl9*). We reveal that RBFOX2 regulates AS in response to obesogenic diets, and that the RBFOX2-regulated AS network can be targeted therapeutically. Splice-switching oligonucleotides (SSOs) modulating *Scarb1* splicing revert the accumulation of lipotoxic species in RBFOX2-deficient mouse hepatocytes, alleviate liver inflammation associated with diet-induced obesity in vivo and promote an anti-atherogenic lipoprotein profile in the blood, demonstrating the potential of isoform-specific RNA therapeutics for metabolic pathologies.

## Results

### Nutrition-promoted changes in the liver splicing machinery

**Fig. 1:**
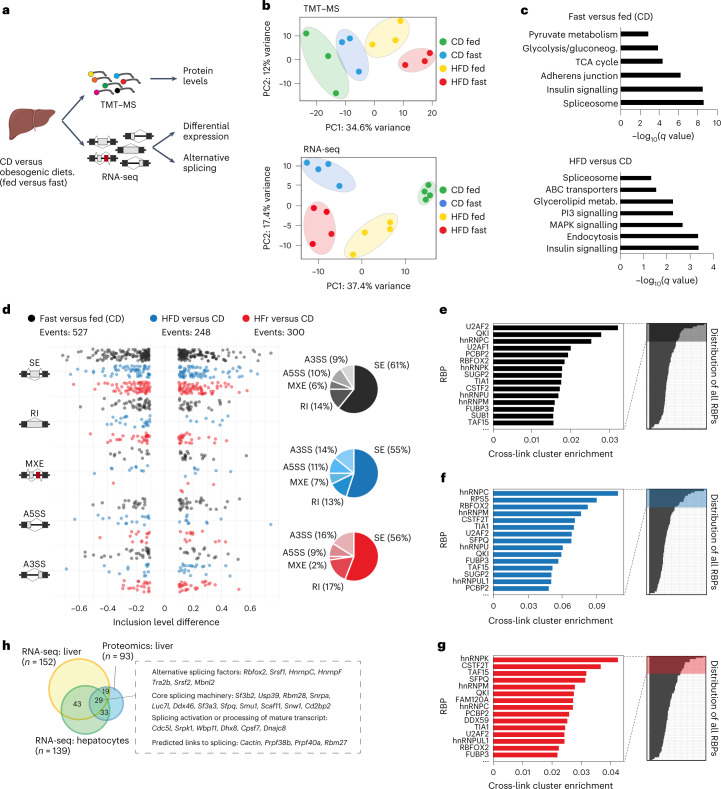
Expression of pre-mRNA splicing machinery is regulated by nutritional inputs in the liver. **a**, Schematic representation of experimental design. Livers from male mice fed a HFD or a CD were harvested in fed (ad libitum) or fasted (16 h) state and processed for either high-throughput TMT–MS (isobaric mass tagging) proteomics (*n* = 3) or RNA-seq analysis (*n* = 4). **b**, Principal component analysis for TMT–MS (upper) and RNA-seq (lower) analyses. **c**, Gene ontology analysis of proteins differentially expressed between fasted versus fed state (upper) and between HFD versus CD livers (lower). **d**, Analysis of differential AS events between CD fast versus fed (black), HFD versus CD (blue) and HFr versus CD (red). Percentage of events changing within each comparison is represented by pie chart (right) (A3SS, alternative 3' splice site; A5SS, alternative 5' splice site; MXE, mutually exclusive exons; RI, retained intron; SE, skipped exon). **e**–**g**, Enrichment of eCLIP cross-linking surrounding conserved AS events, differentially regulated in each comparison, in HepG2 cells from ENCODE database. eCLIP binding enrichment: fast versus fed (**e**), HFD versus CD (**f**) and HFr versus CD (**g**). **h**, Venn diagram showing the splicing factors differentially expressed in HFD versus CD from RNA-seq (yellow) and TMT–MS analyses (blue) and the overlap of splicing factors detected in primary hepatocytes (green).

**Extended Data Fig. 1:**
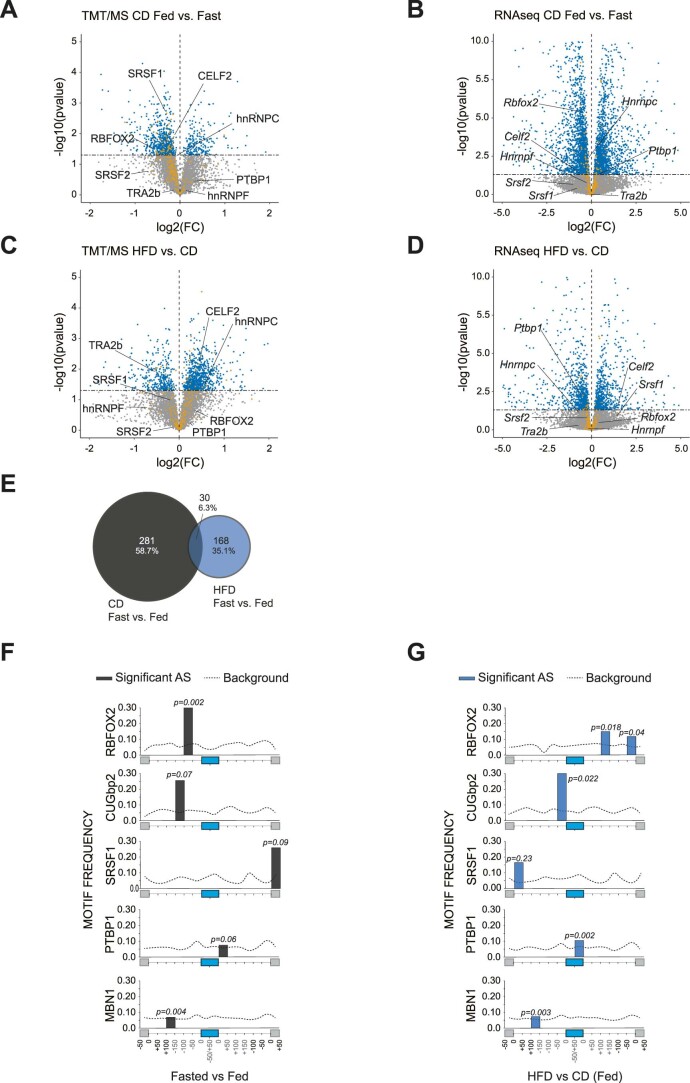
Splicing factors differentially expressed in specific metabolic conditions in the liver. A.- Volcano plot showing splicing factor (gold) expression in liver of mice in fed or fasted state as determined by TMT/MS analysis or B.- RNAseq analysis. C.- Splicing factor expression in mice fed a HFD vs CD as determined by TMT/MS analysis and D.- RNAseq analysis. E.- Overlap between AS events differentially regulated in feeding/fasting cycles in mice fed a CD or a HFD. F.- Splicing factor motif enrichment within and around cassette exons alternatively spliced in the liver of mice in fasted vs fed state and G.- in HFD vs. CD. Enrichment in non-AS exons was used as a background (dot lines).

To investigate liver metabolic plasticity during health and disease, we conducted unbiased analyses of the liver transcriptome (RNA-sequencing, RNA-seq) and proteome (tandem mass tag–mass spectrometry, TMT–MS) of mice fed either control (CD) or high-fat diets (HFD), under both fed and starved conditions (Fig. 1a). Proteomic analysis identified 5,999 proteins in all experimental conditions (Supplementary Table 1). Principal component analysis confirmed the effect of the dietary interventions in the liver proteome (Fig. 1b, top) and transcriptome (Fig. 1b, bottom). Gene ontology analysis showed that feeding and fasting cycles specifically modify the expression of ‘spliceosome’ proteins involved pre-mRNA splicing (false discovery rate (FDR) of 2.43 × 10^−9^), as well as core metabolic categories such as ‘insulin signalling’ and ‘TCA cycle’ (Fig. 1c top and Extended Data Fig. 1a,b). Similarly, expression of spliceosome proteins in the liver was modified by consuming a HFD (FDR = 4.72 × 10^−2^) (Fig. 1c bottom, Extended Data Fig. 1c,d). Thus, components of the pre-mRNA splicing machinery are selectively regulated by metabolic inputs in the liver, suggesting a potential effect on pre-mRNA splicing and/or AS.

Direct analysis of AS profiles by RNA-seq identified significant changes associated with feeding/fasting cycles in mice fed a CD, and with HFD (Fig. 1d). AS changes promoted by HFD included skipped exons, the most abundant category (55%), followed by retained introns (13%), alternative 3′ splice sites (A3SS) (14%), alternative 5′ splice sites (A5SS) (11%) and mutually exclusive exons (MXE) (7%). Increased sugar consumption is a significant contributor to diet-induced liver disease and associated cardiometabolic disease. We investigated AS events promoted by a high-fructose (HFr) diet as an alternative model of diet-induced obesity^16^. AS changes associated with HFr diet included skipped exons, the most abundant AS event identified in these conditions (56%), followed by retained introns (17%), A3SS (16%), A5SS (9%) and MXE (2%), (Fig. 1d). AS changes promoted by feeding or fasting cycles in CD mice were attenuated in diet-induced obesity (Extended Data Fig. 1e) suggesting that perturbation of liver AS networks contributes to the reduced metabolic plasticity observed in obesity. Collectively, these results reveal specific changes in pre-mRNA AS programs in physiological (feeding or fasting cycles) and pathophysiological (HFD- and HFr-induced obesity) adaptations.

### Splicing factor RBFOX2 is modulated by diet in the liver

**Fig. 2:**
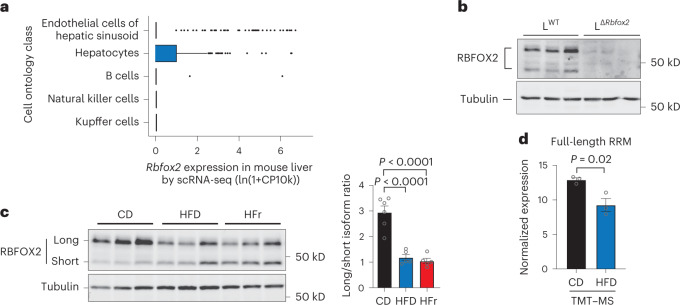
RBFOX2 is a splicing factor expressed in the liver. **a**, Liver single-cell analysis of *Rbfox2* expression. Boxes show interquartile ranges (IQR) with an horizontal bar representing the median of gene counts from all cells in the respective cluster passing the QC cut-off of 500 genes and 1,000 UMI from ref. ^18^. Whiskers represent the upper and lower 1.5× IQR and points denote outliers. Cell numbers per cluster: endothelial cell of hepatic sinusoid (182), hepatocyte (391), B cell (41), natural killer cell (39) and Kupffer cell (61). **b**, Western blot of L^WT^ and L^Δ*Rbfox2*^ liver lysates showing RBFOX2 expression in male hepatocytes (*n* = 3). **c**, Western blot of C57BL6 liver lysates showing RBFOX2 expression in male mice fed a CD, a HFD or a HFr diet (*n* = 6 per condition, image shows three representative samples). Right, quantification of long/short RBFOX2 variants ratio. **d**, Expression of RBFOX2 containing full-length RRM motif as quantified by TMT–MS (*n* = 3). Bar graphs are represented as mean ± s.e.m. Statistical significance was determined by one-way ANOVA and Dunnett’s multiple comparisons test (**c**) or two-tailed unpaired *t*-test (**d**) of biologically independent samples. Source data

**Extended Data Fig. 2:**
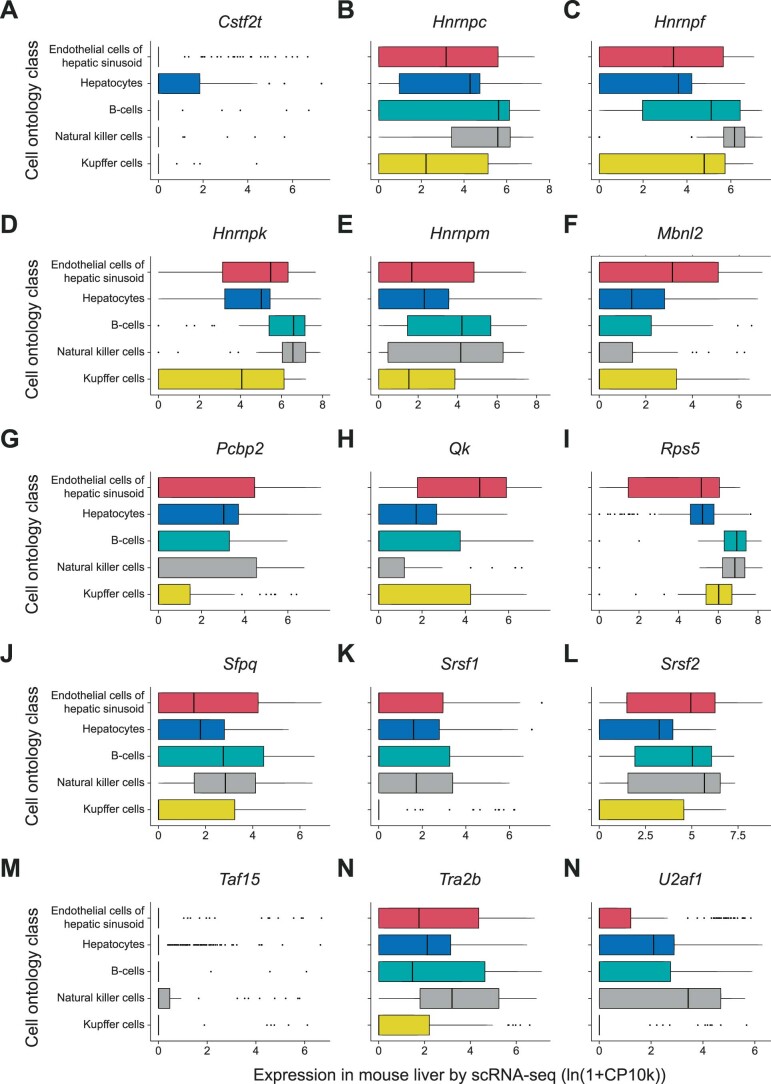
Single-cell RNAseq analysis of RNA binding protein gene expression in the liver. Liver single-cell RNAseq analysis of the expression of relevant AS factors. Boxes show interquartile ranges (IQR) with a horizontal bar representing the median of gene counts from all cells in the respective cluster passing the QC cut-off of 500 genes and 1000 UMI from^18^. Whiskers represent the upper and lower 1.5 x IQR and points denote outliers. Cell numbers per cluster: Endothelial cell of hepatic sinusoid (182), Hepatocyte (391), B cell (41), Natural killer cell (39) and Kupffer cell (61). Source data

To investigate the splicing factors controlling the changes detected in liver AS profiles in different nutritional states, we reasoned such splicing factors should: (1) show detectable expression in liver and/or hepatocytes and (2) have enriched binding at regions within or surrounding the alternatively spliced exons in the liver. We performed an unsupervised motif enrichment analysis of the sequences within and surrounding alternatively spliced exons in physiological (feeding/fasting cycles) and pathological (diet-induced obesity) states in the liver. We observed significant enrichment of splicing factor binding motifs, including RBFOX2, CUGBP2, SRSF1, PTBP1 and MBN1 (Extended Data Fig. 1f,g). Analysis of splicing factors with conserved cross-linking peaks within and surrounding AS exons in human liver cells^17^ identified eight splicing factors (U2AF2, RBFOX2, QKI, hnRNPC, PCBP2, TIA1, hnRNPM and TAF15) as the top 20% ranking factors in all three comparisons analysed: feeding and fasting cycles (Fig. 1e), HFD-induced obesity (Fig. 1f) and HFr-induced obesity (Fig. 1g). We confirmed that RBFOX2 is expressed in the liver (Fig. 1h), and showed that hepatocytes account for most of the *Rbfox2* liver expression, although *Rbfox2* was also detected in endothelial cells, on the basis of the analysis of a mouse single-cell RNA-seq dataset^18^ (Fig. 2a). Other splicing factors potentially involved in AS regulation showed expression across other liver-resident cell populations (Extended Data Fig. 2). We generated *Alb_cre*^*−*^*Rbfox2*^*LoxP/LoxP*^ (L^WT^) and *Alb_cre*^*+*^*Rbfox2*^*LoxP/LoxP*^ (L^Δ*Rbfox2*^*)* mice to inactivate the *Rbfox2* gene selectively in hepatocytes. Western blot analysis showed that RBFOX2 is undetectable in the liver of L^Δ*Rbfox2*^ mice, confirming that hepatocytes account for most of its expression in the liver (Fig. 2b). These results indicate a relevant role for RBFOX2 in regulating AS in hepatocytes.

*Rbfox2* is regulated at the transcriptional level by feeding and fasting cycles in the liver (Extended Data Fig. 1a,b). Alternative promoters and AS can generate multiple RBFOX2 isoforms with different splicing activity^19^, including a dominant negative form having a truncated RNA-recognition motif (RRM). Western blot analysis showed that both HFD- and HFr-diet-induced obesity are associated with decreased expression of the main RBFOX2 isoform in the liver (Fig. 2c). TMT–MS proteomic analysis shows that these changes are associated with decreased levels of full-length active RBFOX2 (Fig. 2d) suggesting a potential modulation of RBFOX2 function in the liver in diet-induced obesity, and that RBFOX2 could coordinate AS changes in response to physiological and pathological metabolic signals.

### RBFOX2 controls cholesterol-regulating genes via AS

**Fig. 3:**
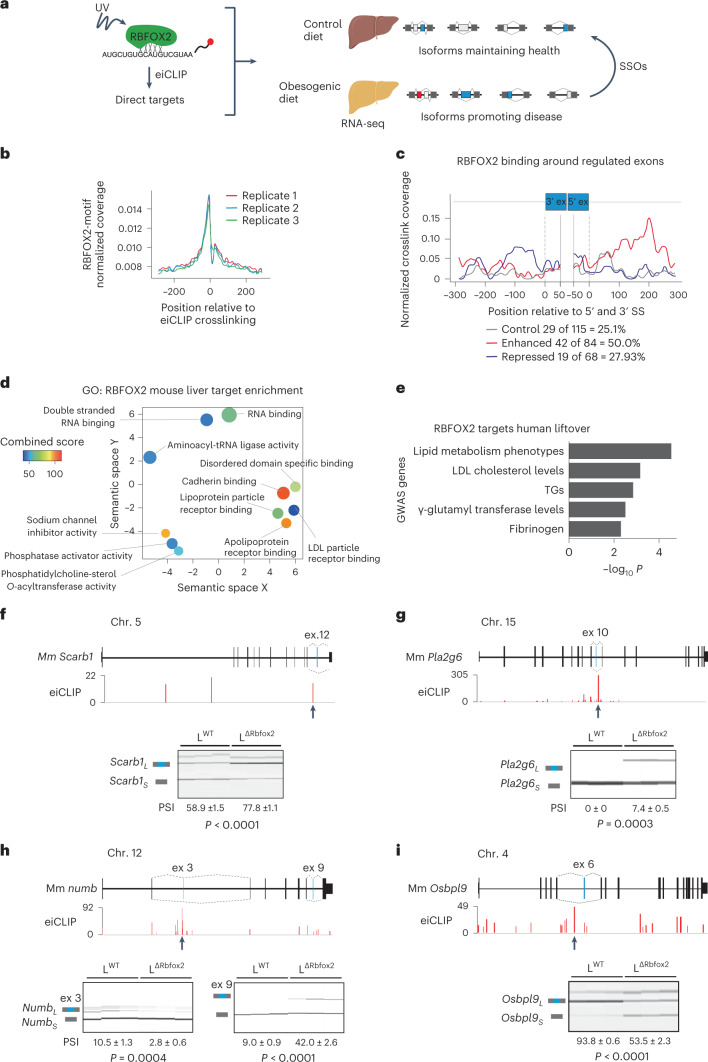
RBFOX2 controls AS in a cluster of lipid-regulatory genes in the liver. **a**, Cartoon depicting the experimental strategy to identify RBFOX2-regulated AS programmes and isoforms for RNA therapeutics. **b**, RBFOX2 motif enrichment relative to eiCLIP-RBFOX2 cross-linking positions in mouse hepatocytes (*n* = 3). **c**, RNA maps showing normalized density of RBFOX2 eiCLIP crosslink sites relative to 5′ and 3′ splice sites (SSs) of selected exons identified by RNA-seq in liver from L^WT^ and L^Δ*Rbfox2*^ mice. **d**, Gene ontology molecular function analysis of RBFOX2 cross-linked genes in mouse liver visualized with REVIGO. Bubble size corresponds to number of combined gene ontology terms. Colour corresponds to combined score. **e**, Genome-wide association study of human genes cross-linked by RBFOX2 in hepatocytes. **f**, Schematic representation of *Scarb1* gene showing RBFOX2 eiCLIP peak location surrounding exon 12 (arrow). Semiquantitative PCR plus capillary electrophoresis analysis of *Scarb1* exon 12 in liver and quantification of AS in L^WT^ and L^Δ*Rbfox2*^ male mice (bottom). **g**, Analysis of RBFOX2 regulation of *Pla2g6* exon 10, **h**,**i**, *Numb* exon 3 (**h**) and exon 9 and (**i**) *Osbpl9* exon 6. PSI values are represented as mean ± s.e.m. (*n* = 6–8). Statistical significance was determined by two-tailed unpaired *t*-test of biologically independent samples. Source data

**Extended Data Fig. 3:**
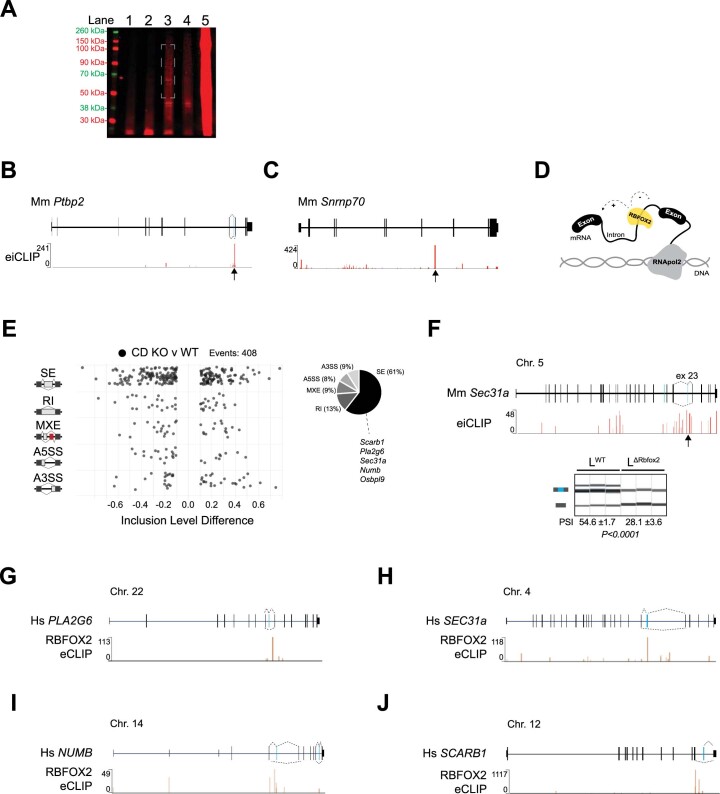
Analysis of RBFOX2-mediated AS regulation in the liver. A.- NuPAGE gel visualising protein-RNA complexes obtained in eiCLIP in hepatocytes. Lanes 1: No antibody, 2: No UV, 3: 0.2U/ml RNase, 4: 0.1U/ml RNase and 5: size-matched input. Region from which protein-RNA complexes were cut from the membrane is marked by a dashed box (n = 3). B.- eiCLIP analysis confirms RBFOX2 crosslink to *Ptbp2* and C.- *Snrnp70* pre-mRNA transcripts. D.- Cartoon depicting positional effect on RBFOX2 regulation of AS. E.- Bubble-plot representing significant AS events between L^Δ*Rbfox2*^ and L^WT^ livers. Significant events are represented by pie chart (right) as a percentage of total events. Selected transcripts with eiCLIP RBFOX2-crosslinking peaks are indicated. F.- eiCLIP track showing RBFOX2 crosslink (top) and semi-quantitative PCR showing that RBFOX2 promotes *Sec31a* exon 23 skipping. PSI values are represented as mean ± s.e.m. (n = 6-8). Statistical significance was determined by two-sided *t*-test of biologically independent samples. G.- RBFOX2 crosslink in human hepatocyte samples to *PLA2G6*, H.- *SEC31A*, I.- *NUMB* and J.- *SCARB1* pre-mRNA transcripts. Source data

Splicing factors frequently regulate AS through interconnected *cis*- and *trans*-mediated effects^20,21^. Identification of direct targets for endogenous splicing factors in the liver has been hampered by rapid pre-mRNA degradation during cross-linking and immunoprecipitation (iCLIP) analysis in liver samples, resulting in very limited information regarding AS programmes in adult liver that maintain or perturb homeostasis. To overcome this problem, we used an enhanced individual-nucleotide-resolution iCLIP (eiCLIP) protocol with an expedited and improved library preparation workflow (Methods) to enhance the recovery of RBFOX2 cross-linked pre-mRNA products (Fig. 3a and Extended Data Fig. 3a). The specificity of the signal was confirmed by peak analysis, showing an enrichment of the RBFOX2 consensus (U)GCAUG binding motif^22^ (Fig. 3b), and direct RBFOX2 targets in hepatocytes included previously described bona fide targets such as *Ptbp2* and *Snrnp70* (ref. ^20^) (Extended Data Fig. 3b,c).

The RBFOX2 protein promotes or represses AS in a position-dependent manner^20,22–27^ (Extended Data Fig. 3d). Analysis of RBFOX2 cross-linking positions upstream or downstream of AS exons (identified by RNA-seq in L^WT^ and L^Δ*Rbfox2*^ mice; Extended Data Fig. 3e) showed that, in the liver, this positional effect is more robust in enhanced exons (50.0%) than in repressed exons (27.9%) compared to control exons (25.1%) (Fig. 3c).

Gene ontology analysis showed that RBFOX2 cross-linked clusters are highly enriched in transcripts encoding for proteins involved in phosphatidylcholine-sterol-*O*-acyltransferase activity (adjusted *P* value (*P*) = 3.66 × 10^−2^), lipoprotein particle receptor binding (adjusted *P* = 1.13 × 10^−2^), apolipoprotein receptor binding (adjusted *P* = 2.61 × 10^−^^3^) and low-density lipoprotein (LDL) particle receptor binding, suggesting a role for RBFOX2 in controlling lipid metabolism (Fig. 3d). Other targets are involved in functions including cadherin binding (adjusted *P* = 7.42 × 10^−16^), disordered domain specific binding (adjusted *P* = 2.72 × 10^−4^) and also RNA-binding proteins (adjusted *P* = 3.72 × 10^−^^14^), consistent with RBFOX2 modulating additional layers of transcriptional regulation^20^. The list of RBFOX2 targets contributing to each gene ontology category are included in Supplementary Table 2.

The human orthologous genes of the mouse RBFOX2 targets detected by eiCLIP are enriched in genes implicated by genome-wide association study in human lipid metabolism phenotypes, LDL cholesterol levels or triglycerides (TGs) (Fig. 3e). These include the *Scarb1* gene, which encodes the class B scavenger receptor SR-BI, a high-density lipoprotein (HDL) receptor that mediates cholesterol uptake and modifies plasma HDL and bile cholesterol^28,29^, *Pla2g6* that is a phospholipase A2 group VI^30^, *Sec31a*, a core component of the COPII vesicle trafficking system involved in sterol regulatory element-binding protein 1 (SREBP1) activation^31^ and processing of ApoB-containing lipoprotein^32^, the oxysterol-binding protein *Osbpl9* and *Numb*, an adaptor protein involved in clathrin-dependent reverse cholesterol transport from bile^33^.

eiCLIP profiles at the *Scarb1* pre-messenger RNA (mRNA) transcript revealed that RBFOX2 binds upstream of exon 12. PCR analysis of the livers of L^Δ*Rbfox2*^ mice confirmed that RBFOX2 promotes *Scarb1* exon 12 skipping (Fig. 3f). RBFOX2 also promotes skipping or inclusion of specific exons in *Pla2g6* (Fig. 3g)*, Numb* (Fig. 3h)*, Osbpl9* (Fig. 3i) and *Sec31a* (Extended Data Fig. 3f). Analysis of RBFOX2 binding in human hepatoma cells confirmed the conservation of RBFOX2 cross-linking within alternatively spliced exons in the orthologous human transcripts (Extended Data Fig. 3g–j). Thus, RBFOX2 directly regulates the AS of a network of genes involved in lipid homeostasis in the liver.

### RBFOX2 regulates cholesterol metabolism in a lipogenic diet

**Fig. 4:**
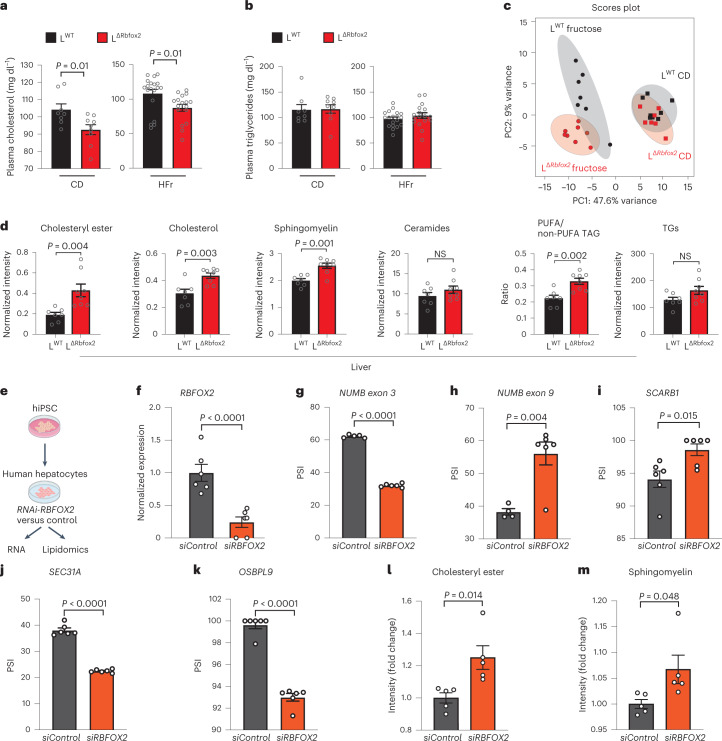
RBFOX2 controls lipid homeostasis in the liver. **a**,**b**, Plasma lipid analysis showing total cholesterol levels (**a**) and TG (**b**) in L^WT^ and L^Δ*Rbfox2*^ male mice fed a CD (*n* = 8–9) and a HFr diet (*n* = 18–19). **c**, PCA plot of liver lipid profiles of L^WT^ and L^Δ*Rbfox2*^ mice fed a CD and a HFr determined by LC–MS. **d**, LC–MS lipidomic analysis showing total levels of indicated species normalized to tissue mass and PUFA/non-PUFA TG ratio (*n* = 7–8). **e**, Cartoon depicting strategy for iPSC-derived human hepatocyte analysis. **f**, RT–qPCR analysis showing knockdown of *RBFOX2* in human hepatocytes (*n* = 6). **g**–**k**, Analysis of RBFOX2-mediated regulation of *NUMB* exon 3 (**g**), *NUMB* exon 9 (**h**), *SCARB1* exon 12 (**i**), *SEC31A* exon 21 (**j**) and *OSBPL9* exon 6 (**k**). PSI values are represented as mean ± s.e.m. (*n* = 4–6). **l**,**m**, Lipidomics quantification of the cholesteryl ester (**l**) and sphingomyelin accumulation (**m**) on RBFOX2 knockdown in human hepatocytes (*n* = 5). Results are represented as mean ± s.e.m. Statistical significance was determined by two-tailed unpaired *t*-test of biologically independent samples. NS, not significant. Source data

**Extended Data Fig. 4:**
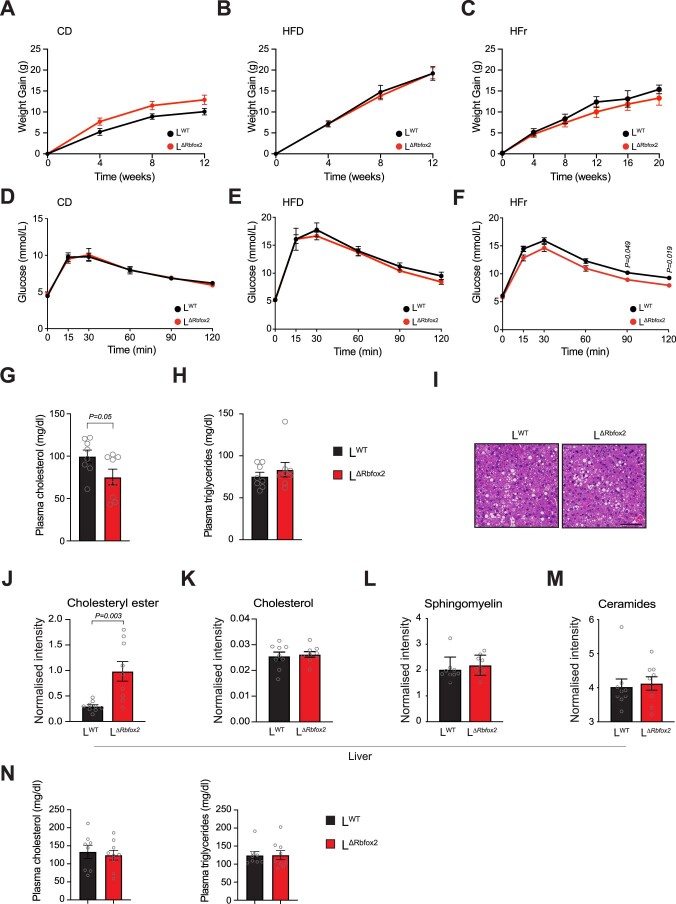
RBFOX2 is involved in the regulation of cholesterol homeostasis. A.- Body-weight gain over time of L^WT^ and L^Δ*Rbfox2*^ animals fed a CD, (B.-) a HFD and (C.-) a HFr diet. D.- Glucose tolerance test of L^WT^ and L^Δ*Rbfox2*^ fed a CD, (E.-) a HFD and (F.-) a HFr diet. G.- Blood analysis of L^Δ*Rbfox2*^ vs. L^WT^ female mice showing cholesterol levels, and H.- triglycerides. I.- H&E staining of liver from L^Δ*Rbfox2*^ vs. L^WT^ mice fed a HFr diet. Scale bar 50 µm. J-M.- LC-MS/MS analysis of the specified lipids in the liver of L^Δ*Rbfox2*^ vs. L^WT^ mice fed a HFD. N.- Blood cholesterol and triglyceride levels in L^Δ*Rbfox2*^ vs. L^WT^ mice fed a HFD. Results are represented as mean ± SEM (n = 8-9). Statistical significance was determined by two-way ANOVA with Sidak’s multiple correction test (A-F) or by two-tailed unpaired *t*-test (G-N) of biologically independent samples. Source data

L^Δ*Rbfox2*^ mice are viable and are born at expected Mendelian ratios. When fed control chow, a HFD diet or a HFr diet, L^Δ*Rbfox2*^ mice did not show significant changes in body weight (Extended Data Fig. 4a–c) or glucose tolerance (Extended Data Fig. 4d–f) suggesting that RBFOX2 ablation does not interfere with normal liver development. In contrast to the normal glucose homeostasis, blood lipid profile analysis revealed that L^Δ*Rbfox2*^ male mice showed a significant decrease in total cholesterol (Fig. 4a) but not in TGs (Fig. 4b), when consuming a HFr diet. Similar results were obtained when studying L^WT^ and L^Δ*Rbfox2*^ female mice (Extended Data Fig. 4g,h).

The liver is central to lipid homeostasis. To investigate the metabolic changes associated with the altered blood lipid profile, livers from L^WT^ and L^Δ*Rbfox2*^ mice were analysed by untargeted liquid chromatography–mass spectrometry (LC–MS) lipidomics. Principal component analysis showed that the HFr diet was associated with marked changes in the overall metabolomic profile compared to CD samples (Fig. 4c). In mice fed a HFr diet, ablation of RBFOX2 was associated with an increase in hepatic lipid content, particularly cholesteryl esters, cholesterol and sphingomyelins (Fig. 4d). While we did not observe differences in steatotic patterns (Extended Data Fig. 4i), we noted a change in TG composition favouring longer polyunsaturated fatty acyl (PUFA) chains (as denoted by the PUFA/non-PUFA TG ratio) in L^Δ*Rbfox2*^ mice (Fig. 4d). When fed a HFD, L^Δ*Rbfox2*^ mice also showed an increase in intrahepatic total cholesteryl esters. However, unlike mice fed a HFr diet, no overall changes in cholesterol or sphingomyelins (Extended Data Fig. 4j–m), nor changes in blood cholesterol were observed (Extended Data Fig. 4n), suggesting a partial phenotype that is exacerbated when L^Δ*Rbfox2*^ mice are fed a pro-lipogenic HFr diet.

**Extended Data Fig. 5:**
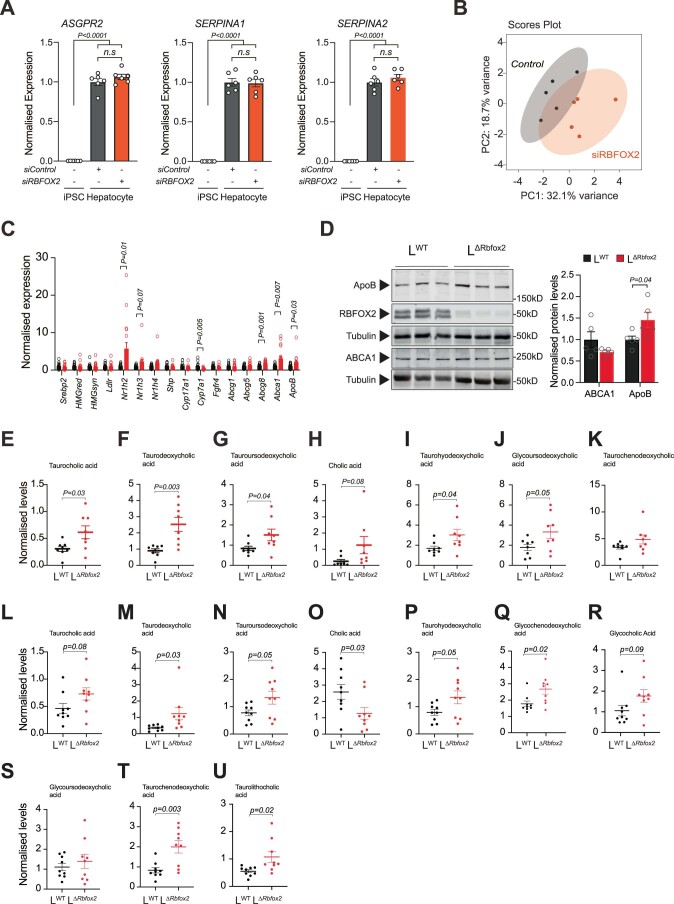
RBFOX2 regulates lipid metabolism in human hepatocytes. A.- RT-qPCR analysis of *ASGPR2*, *SERPINA1* and *SERPINA2* in human hepatocytes upon *RBFOX2* targeted knockdown (n = 6) B.- PCA plot of lipidomic analysis of human hepatocytes upon *RBFOX2* targeted knockdown as determined by LC-MS. (n = 5). C**.-** RT-qPCR analysis of genes involved in cholesterol and bile acid homeostasis, in the liver of L^Δ*Rbfox2*^ and L^WT^ mice fed a HFr diet (n = 18-20). D.- Representative western-blot showing APOB and ABCA1 expression in the liver of L^ΔRbfox2^ vs. L^WT^ mice (n = 5). E-K.- Bile acids levels in the liver of L^Δ*Rbfox2*^ vs. L^WT^ mice fed a HFr diet or (L-U) a HFD diet as determined by LC-MS/MS. Samples were normalised by tissue weight and internal standard (n = 8). Results are represented as mean ± s.e.m.; Statistical significance was determined by one-way ANOVA with Tukey’s multiple comparisons test (A) or two-tailed unpaired *t*-test (C-U) of biologically independent samples. Source data

CLIP analysis with sequencing suggested RBFOX2 targets are conserved in humans (Extended Data Fig. 3g–j). To determine whether RBFOX2 is involved in lipid metabolism in human hepatocytes, we used induced pluripotent stem cell (iPSC)-derived hepatocytes and RNA interference (RNAi) to silence *RBFOX2* (Fig. 4e), which we confirmed by quantitative PCR (qPCR) (Fig. 4f). RBFOX2 silencing did not affect the expression of mature hepatocyte/differentiation markers such as *ASGPR2*, *SERPINA1* and *SERPINA2* (Extended Data Fig. 5a). Consistent with human RBFOX2 binding to pre-mRNA, the effect of *RBFOX2* deficiency in AS of *SCARB1*, *SEC31A*, *OSBPL9*, *PLA2G6* and *NUMB* is maintained in human hepatocytes (Fig. 4g–k), supporting conservation of this regulatory network. Moreover, lipidomic analysis of human hepatocytes showed that *RBFOX2* silencing promotes changes in lipid composition (Extended Data Fig. 5b) including cholesteryl esters and sphingomyelins accumulation (Fig. 4l,m). These results support the role of RBFOX2 in controlling a conserved AS network involved in lipid metabolism.

The simultaneous accumulation of cholesterol in the liver of L^Δ*Rbfox2*^ mice and the decrease in cholesterol in the plasma suggested that RBFOX2 plays a specific role in the regulation of hepatic cholesterol uptake, trafficking and/or efflux. To further understand these mechanisms, we used RNA-seq to compare the transcriptome of L^WT^ and L^Δ*Rbfox2*^ mice fed a HFr diet. RBFOX2 ablation in the liver led to transcriptional changes in cholesterol biosynthesis (ratio 0.138; *P* = 1.7 × 10^−^^5^), the mevalonate pathway (ratio 0.214; *P* = 5.25 × 10^−^^5^), the liver X receptor (LXR) pathway (ratio 0.02; *P* = 2.75 × 10^−^^2^) and the farnesoid X receptor pathway (ratio 0.02; *P* = 3.09 × 10^−2^), consistent with a role of RBFOX2 in lipid and cholesterol metabolism. Changes were also found in oxidative phosphorylation (ratio 0.138; *P* = 2.51 × 10^−^^17^) and mitochondrial dysfunction (ratio 0.0877; *P* = 2.51 × 10^−^^14^).

Cholesterol metabolism is controlled by a feedback mechanism involving SREBPs, an ER-resident family of transcription factors^34^. An ad hoc qPCR analysis of genes involved in cholesterol homeostasis showed that increased intrahepatic cholesterol is not associated with increased expression of key biosynthetic genes, including *Srebf2*, *Hmgcs* and *Hmgcr* (Extended Data Fig. 5c), suggesting that this feedback regulation remained intact. Increases in the expression of *Abca1*, *Abcg8* and *Nr1h2* (LXR beta) and *Nr1h3* (LXR alpha) further suggested a compensatory increase in cholesterol efflux pathways caused by cholesterol accumulation on RBFOX2 deficiency (Extended Data Fig. 5c). An increase in ApoB protein was also observed, although the difference between L^WT^ and L^Δ*Rbfox2*^ for ABCA1 was not statistically significant (Extended Data Fig. 5d).

Reverse HDL-cholesterol uptake and conversion into bile acids in the liver is a main pathway for cholesterol excretion through the bile. We proposed that decreased cholesterol in blood of L^Δ*Rbfox2*^ mice and simultaneous increase in intrahepatic cholesterol could lead to an increase in bile acid levels. Consistent with this hypothesis, LC–tandem MS (LC–MS/MS) analysis showed an increase in bile acids, including taurocholic, taurodeoxycholic, tauroursodeoxycholic and cholic acids (Extended Data Fig. 5e–k). When fed a HFD, L^Δ*Rbfox2*^ mice also showed an increase in specific intrahepatic bile acids, compared to L^WT^ control mice (Extended Data Fig. 5l–u).

**Extended Data Fig. 6:**
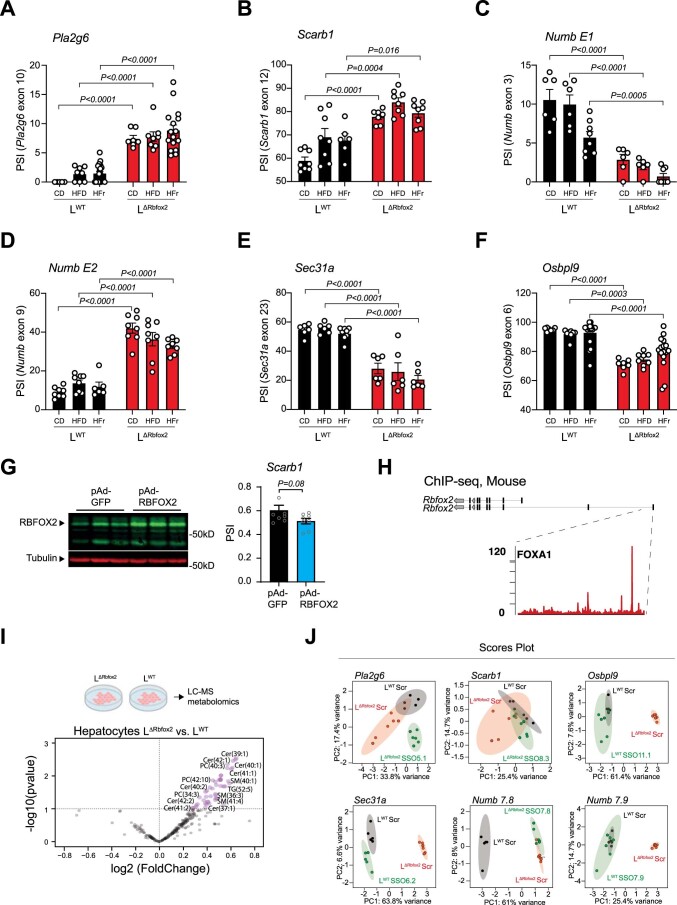
Quantification of AS of direct RBFOX2 targets in mice fed a CD, a HFD or a HFr diet. A.- Quantification of percentage splice in (PSI) for *Pla2g6* (exon 10), (B.-) *Scarb1* (exon 12), (C.-) *Numb* (exon 3), (D.-) *Numb* (exon 9), (E.-) *Sec31a* (exon 23) and (F.-) *Osbpl9* (exon 6) in L^WT^ and L^Δ*Rbfox2*^ mice fed a CD, a HFD or a HFr diet (n = 7-10). G.- Representative western-blot showing RBFOX2 expression levels in the liver of mice after transduction with pAd-RBFOX2 or pAd-GFP control (left) and quantification by PCR/capillary electrophoresis of percentage splice in (PSI) for *Scarb1* (exon 12) (right) (n = 7-9). H.-ChIP-seq signal for FOXA1 in mouse *Rbfox2* promoter in liver. I.- Volcano-plot showing LC-MS lipidomic analysis of L^Δ*Rbfox2*^ *vs* L^WT^ hepatocytes normalised to internal standard and total cell counts (n = 5-6). J.- PCA plot of L^Δ*Rbfox2*^ vs. L^WT^ hepatocytes and relevant SSO treatment as determined by LC-MS lipidomic analysis. Results are represented as mean ± s.e.m. Statistical significance was determined by two-way ANOVA with Tukey’s multiple comparisons test (A-F) or two-tailed unpaired *t*-test (G) of biologically independent samples. Source data

We proposed that changes in RBFOX2 activity could modulate this downstream AS network in response to diet. To test this idea, we analysed AS changes in *Scarb1*, *Pla2g6* and *Numb* in L^WT^ and L^Δ*Rbfox2*^ mice fed a control or an obesogenic diet. Consistent with our hypothesis, HFr promoted significant AS changes in L^WT^ mice and L^Δ*Rbfox2*^ mice failed to induce obesity-specific AS events at *Pla2g6* (Extended Data Fig. 6a)*, Scarb1* (Extended Data Fig. 6b) and *Numb* (Extended Data Fig. 6c). Analysis of other targets such as *Sec31a* and *Osbpl9* confirmed regulation by RBFOX2 (Extended Data Fig. 6e,f), however, the effect of the diet was not detected, suggesting that other factors play a role in the regulation of these genes under obesogenic conditions.

**Fig. 5:**
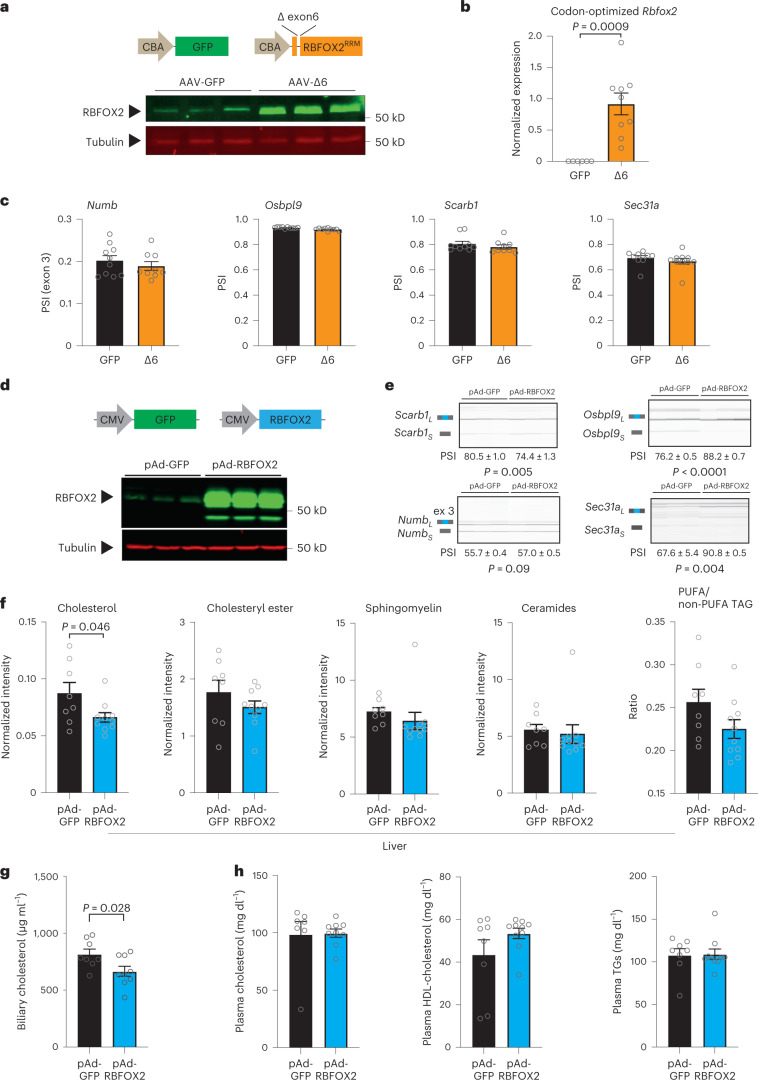
Viral-mediated over-expression of RBFOX2-Δ6 and RBFOX2 WT in the liver. **a**, Cartoon depicting the AAV backbones used to over-express RBFOX2-Δ6 (with a truncated RNA-binding motif RRM) or control green fluorescent protein (GFP) in the liver (top) and representative western blot showing expression levels in the liver of male mice (*n* = 3). **b**, RT–qPCR expression analysis of codon-optimized RBFOX2-Δ6 in the liver (*n* = 6–9). **c**, Quantification of PSI for *Numb* (exon 3), *Osbpl9* (exon 6), *Scarb1* (exon 12) and *Sec31a* (exon 23) (*n* = 9–10). **d**, Cartoon depicting the adenoviral backbones used to over-express RBFOX2 wild type or control GFP (top) and representative western blot showing expression levels in hepatocytes (*n* = 3). **e**, Capillary electrophoresis and quantification of PSI for *Numb* (exon 3), *Osbpl9* (exon 6), *Scarb1* (exon 12) and *Sec31a* (exon 23) after RBFOX2 over-expression in hepatocytes (*n* = 6). **f**, LC–MS lipidomic analysis showing total levels of free cholesterol, cholesteryl ester, sphingomyelin and ceramide normalized to liver tissue mass and PUFA/non-PUFA TG ratio in male mice fed a HFr diet after transduction with pAd-RBFOX2 or pAd-GFP control (*n* = 8–9). **g**, Cholesterol levels quantified in the bile of mice fed a HFr diet after transduction with pAd-RBFOX2 or pAd-GFP control (*n* = 8–9). **h**, Plasma lipid analysis showing total cholesterol, HDL cholesterol and TGs in mice fed a HFr diet after transduction with pAd-RBFOX2 or pAd-GFP control (*n* = 8–9). Results are represented as mean ± s.e.m. Statistical significance was determined by two-tailed unpaired *t*-test of biologically independent samples. Source data

To further analyse how RBFOX2 regulates this AS network we asked whether AS changes are associated with the decreased levels of active RBFOX2 or increased levels of the inactive (lacking RRM motif) protein (Fig. 2c,d). We generated an adeno-associated virus (AAV) vector expressing RBFOX2 lacking the RRM motif (RBFOX2-Δ6) (Fig. 5a,b). Over-expression of RBFOX2-Δ6 did not mimic the AS changes associated with RBFOX2 inactivation in mouse and human hepatocytes (Fig. 5c). A vector over-expressing full-length active RBFOX2 (Fig. 5d, Extended Data Fig. 6G) promoted AS changes in the opposite direction to RBFOX2 deficiency (Fig. 5e, Extended Data Fig. 6G), suggesting that the levels of active RBFOX2 are more relevant for AS regulation in the liver than the expression of the dominant negative form.

LC–MS/MS lipidomics showed that RBFOX2 over-expression is associated with a decrease in cholesterol in the liver (Fig. 5f) and in the bile (Fig. 5g). The transient over-expression of RBFOX2 was associated with a mild increase in blood HDL cholesterol that did not reach statistical significance (Fig. 5h). These results confirm that RBFOX2 regulates a network of genes involved in lipid metabolism, and changes in full-length RBFOX2 expression modify cholesterol distribution under a lipogenic diet.

### Liver expression of RBFOX2 is controlled by FOXA1/2

**Fig. 6:**
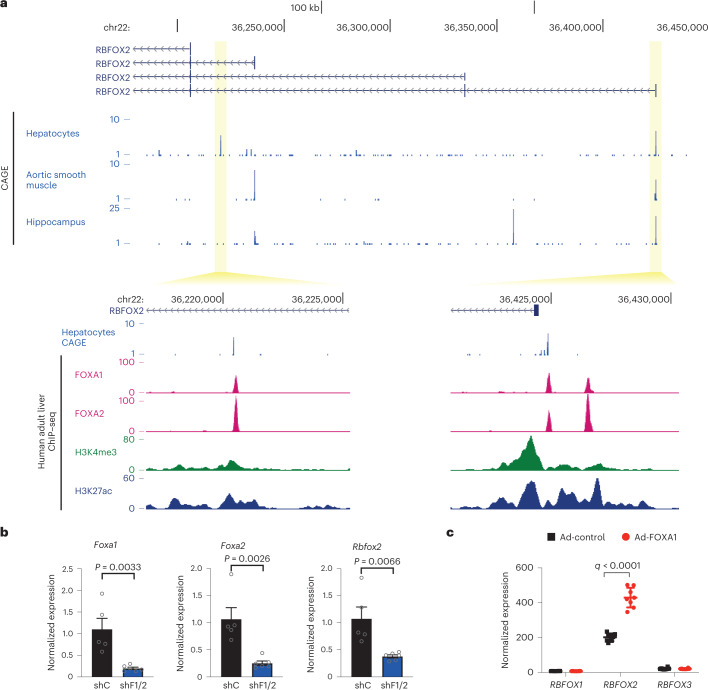
Transcriptional regulation of *Rbfox2* in the liver. **a**, CAGE-detected transcriptional start site (TSS) signal at the promoters of *RBFOX2* transcripts in hepatocytes, aortic smooth muscle and hippocampus. Two transcript isoforms are shown with their respective promoters. Tag clusters are magnified and ChIP–seq signal for FOXA1, FOXA2, H3K4me3 and H3K27ac are shown. **b**, RT–qPCR of *Foxa1, Foxa2* and *Rbfox2* in Hepa1-6 cells expressing a scramble shRNA (shC) or shRNA against Foxa1/2 (shF1/2) (*n* = 5–6). **c**, Microarray analysis of HepG2 cells with adenoviral FOXA1 over-expression (*n* = 8) (GSE30447)^36^. Results are represented as mean ± s.e.m. Statistical significance was determined by two-tailed unpaired *t*-test of biologically independent samples. Source data

To characterize the upstream regulators of *RBFOX2* in human liver, we used FANTOM5 CAGE datasets of transcription initiation sites, as the *RBFOX2* gene has a complex architecture with multiple promoters^19^ (Fig. 6a). Expression of *RBFOX2* in the human hippocampus or aortic smooth muscle is driven by one proximal and one distal promoter, whose activity levels are similar. In hepatocytes, the distal promoter has predominant control over *RBFOX2* expression (threefold change compared to the proximal promoter). CAGE data detected a third promoter in human hepatocytes that does not overlap with previously annotated *RBFOX2* promoters (Fig. 6a). This and the common distal promoter account for most of the *RBFOX2* transcription in human hepatocytes. Both show enrichment of H3K4me3 and H3K27ac by chromatin immunoprecipitation with sequencing (ChIP–seq) in adult human liver, consistent with their role as active hepatic promoters.

Analysis of liver ChIP–seq datasets showed that the *RBFOX2* promoters are bound by FOXA1/2 in humans (Fig. 6a, bottom insets) and mouse (Extended Data Fig. 6h). FOXA1/2 are winged helix transcription factors implicated in bile acid metabolism and protection from liver cholestasis^35^. To validate the role of FOXA1/2 in *Rbfox2* regulation, we knocked down *Foxa1* and *Foxa2* in mouse hepatoma Hepa1-6 cells, which led to a significant decrease in *Rbfox2* expression (Fig. 6b). Conversely, FOXA1 over-expression in HepG2 cells^36^ was associated with a significant increase in *RBFOX2* expression, but not *RBFOX1/3* (Fig. 6c).

### *Scarb1* is an RBFOX2 target with therapeutic potential

To explain the molecular mechanisms underlying the role of RBFOX2 in lipid homeostasis, we designed SSOs to regulate AS of RBFOX2 downstream targets. The efficacy of the SSOs was tested by PCR followed by capillary electrophoresis. SSOs showing potent activity for each splicing event were further used in lipidomic analyses in primary hepatocytes (Supplementary Table 6). LC–MS metabolomics revealed that RBFOX2-deficient hepatocytes have increased lipid accumulation (Extended Data Fig. 6i), confirming the role for RBFOX2 in lipid metabolism in hepatocytes. While SSO7.9, promoting changes in *Numb* exon 3, was not associated with significant lipid remodelling (Extended Data Fig. 6j), SSOs targeting *Numb* exon 9, *Sec31* exon 23, *Pla2g6* exon 10, *Osbpl9* exon 6, and *Scarb1* exon 12 were associated with specific changes in lipid composition (Extended Data Fig. 6j), suggesting that these isoforms mediate the effect of RBFOX2.

**Extended Data Fig. 7:**
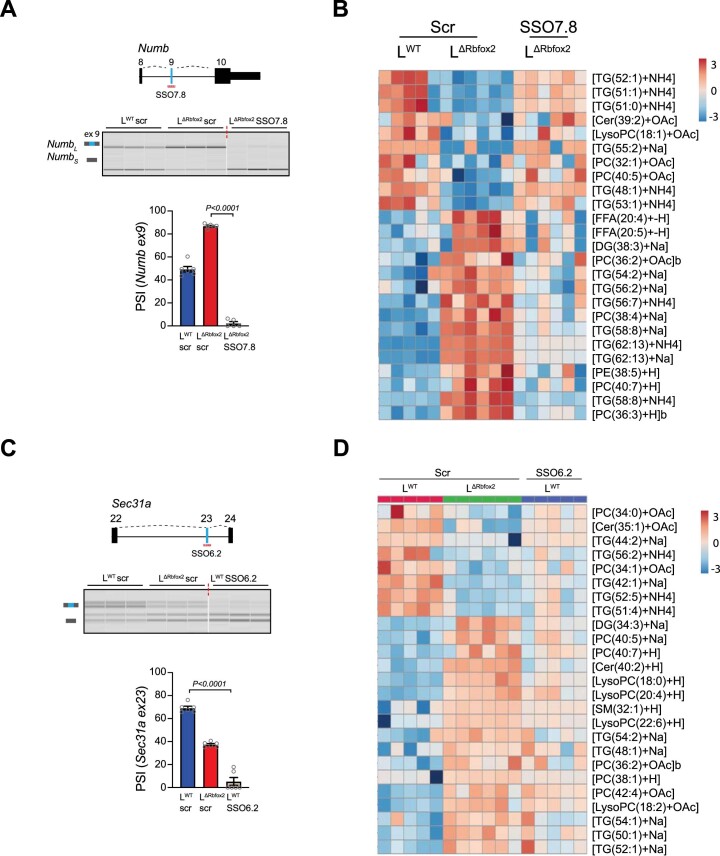
Role of RBFOX2-downstream targets in lipid metabolism in hepatocytes. A.- Capillary electrophoresis and quantification of percentage splice in (PSI) for *Numb* (exon 9), in L^WT^ and L^Δ*Rbfox2*^ hepatocytes upon treatment with SSO7.8 or Scr control. B.- Heatmap showing LC-MS metabolomic analysis of L^WT^ and L^Δ*Rbfox2*^ hepatocytes treated with SSO7.8 or Scr. C.- Capillary electrophoresis and quantification of percentage splice in (PSI) for *Sec31a* (exon 23), in L^WT^ and L^Δ*Rbfox2*^ hepatocytes upon treatment with SSO6.2 or Scr control. D.- Heatmap showing LC-MS metabolomic analysis of L^WT^ and L^ΔRbfox2^ hepatocytes treated with SSO6.2 or Scr. Results are represented as mean ± s.e.m. Statistical significance was determined by one-way ANOVA with Tukey’s multiple comparisons test of biologically independent samples (n = 5-6). Source data

**Extended Data Fig. 8:**
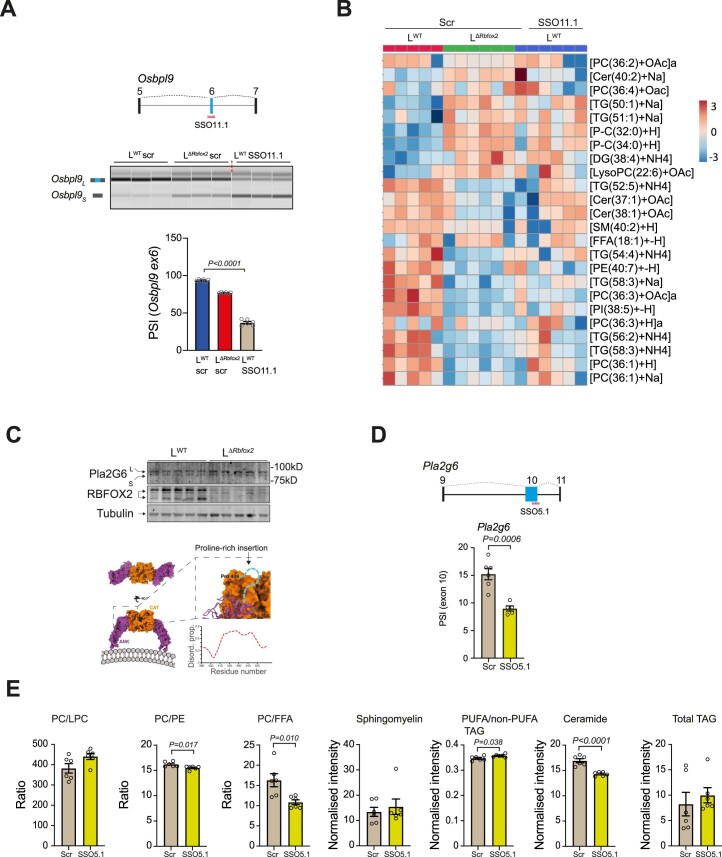
Role of *Osbpl9* and *Pla2g6* isoforms in lipid metabolism. A.- Capillary electrophoresis and quantification of percentage splice in (PSI) for *Osbpl9* (exon 6), in L^WT^ and L^Δ*Rbfox2*^ hepatocytes upon treatment with SSO11.1 or Scr control. Statistical significance was determined by one-way ANOVA with Tukey’s multiple comparisons test of biologically independent samples (n = 5-6). B.- Heatmap showing LC-MS metabolomic analysis of L^WT^ and L^ΔRbfox2^ hepatocytes treated with SSO11.1 or Scr. C.- Western blot showing PLA2G6 expression in wild type and RBFOX2-deficient hepatocytes (top). Cryo-EM maps of PLA2G6 dimers showing tight interaction of the catalytic domains (orange) of each monomer with ankyrin repeats (purple) oriented outward from the core. Ankyrin repeats face ‘claw-like’ towards membrane phospholipids. Insert: 90-degree rotation of the structure detailing the region corresponding to exon 10 of *Pla2g6*_*L*_, a 55-amino intrinsically disordered proline-rich region at the interface of the ankyrin repeats and the catalytic domain. D.- Diagram showing design of splice-switching oligos targeting *Pla2g6* alternative splicing at exon 10. SSO5.1 is designed to promote exon skipping (top) as validated by semi-quantitative PCR analysis (bottom). E.- Quantification of the effect of SSO5.1 on the described lipid species as determined by LC-MS normalised to internal standard and total cell counts. Results are represented as mean ± s.e.m. (n = 5-6). Statistical significance was determined by two-sided unpaired *t*-test of biologically independent samples. Source data

SSO-induced skipping of *Numb* exon 9 (Extended Data Fig. 7a) reverted the accumulation of a number of lipid species, including phospholipids PC (36:3), PC (40:7), PE (38:5), PC (38:4), PC (36:2), PC (40:5), TGs such as TG (58:8), TG (62:13), TG (56:7), TG (56:2) or TG (54:2), and others (Extended Data Fig. 7b). Expression of the short *Sec31a* isoform (skipping exon 23) is associated with increased levels of lipid species such as PC (36:2), PC (42:4), PC (38:1), TG (52:1), DG (34:3) or lysoPC (18:2) and lysoPC (22:6) (Extended Data Fig. 7c,d). Expression of the short *Osbpl9* isoform (skipping of exon 6) in wild type cells promoted an increase in species such as Cer (40:2), TG (50:1), TG (51:1), PC (32:0), PC (34:0) and DG (38:4) resembling RBFOX2-deficient hepatocytes and suggesting a previously uncharacterized role in lipid metabolism (Extended Data Fig. 8a,b). Expression of the long *Pla2g6* isoform including exon 10 (Extended Data Fig. 8c,d) was associated with minor changes in lipid composition (Extended Data Fig. 8e).

**Extended Data Fig. 9:**
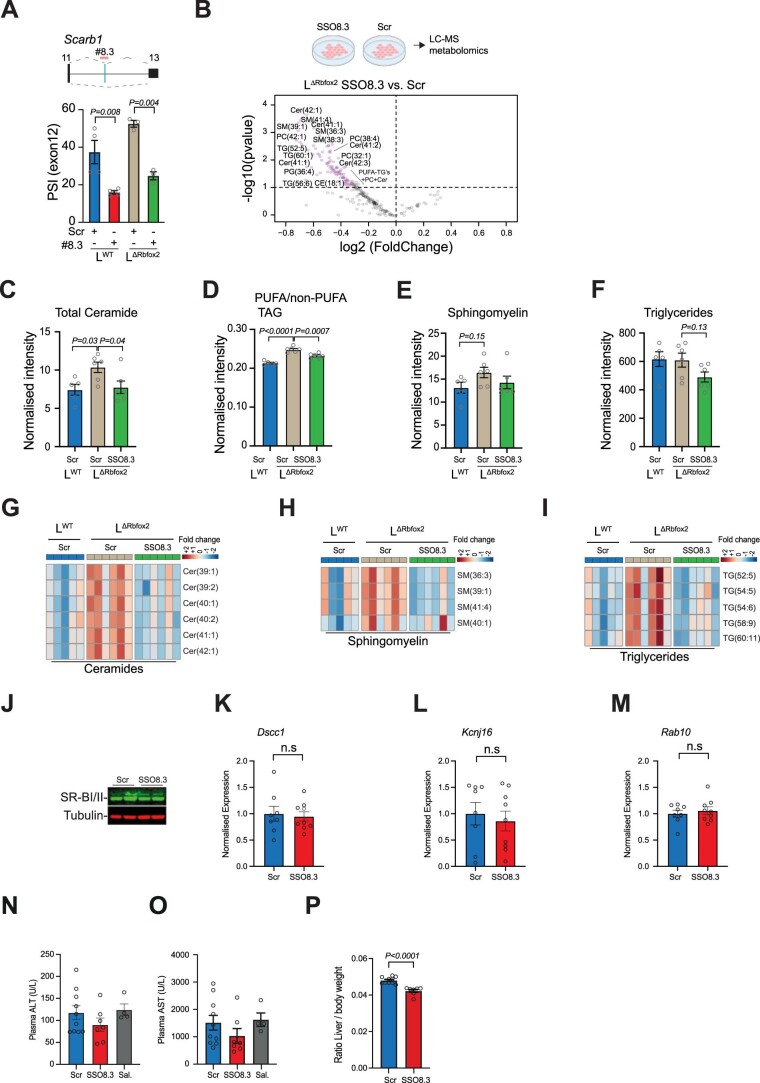
Role of *Scarb1* splicing variants in lipid metabolism. A.- Splice-switching oligonucleotide (SSO8.3) promotes skipping of *Scarb1* exon 12 in primary hepatocytes as determined by semi-quantitative PCR analysis (n = 3-4). B.- Volcano-plot showing LC-MS lipidomic analysis of L^Δ*Rbfox2*^ hepatocytes treated with 100 nM SSO8.3 or Scr for 16 h (n = 5-6). C.- Metabolomics analysis showing levels of total ceramide, D.- PUFA/non-PUFA TG ratio, E.- total sphingomyelin, and F.- total triglycerides (n = 5-6). G-I.- Heatmap showing LC-MS metabolomic analysis of L^WT^ and L^Δ*Rbfox2*^ hepatocytes treated with SSO8.3 or Scr. Intensities were normalised to internal standard and total cell counts. J.- Western-blot showing SR-BI/II levels in the liver after SSO8.3 treatment (n = 2). K.- RT-qPCR expression analysis in liver of mice treated with SSO8.3 or Scr of potential off-targets genes such as *Dscc1*, (L.-) *Kcnj16* and (M.-) *Rab10*. N.- Effect of SSO8.3 injection on circulating ALT and O.- AST levels. P.- Liver/body weight ratio upon SSO8.3 injection (n = 7-10). Results are represented as mean ± s.e.m. Statistical significance was determined by one-way ANOVA or two-sided unpaired *t*-test of biologically independent samples. Source data

Inclusion of *Scarb1* exon 12 generates the canonical SR-BI isoform, while skipping this exon generates an alternative receptor variant with a different adaptor carboxy-terminal domain, named SR-BII^37^. SR-BI/II, is a scavenger receptor for multiple ligands, including very low-density lipoprotein (VLDL) and high-density lipoproteins (HDL), involved in the transport of cholesterol, cholesteryl esters, phospholipids PC, sphingomyelins, lysoPC and other lipid species^38^. However, the overall impact on liver lipidome and the pathophysiological significance of these splicing variants has not been established. SSO8.3 showed significant activity in repressing exon 12 inclusion in primary hepatocytes (Extended Data Fig. 9a). LC–MS analysis showed that this treatment reverted some of the changes associated with RBFOX2 deficiency in hepatocytes (Extended Data Fig. 9b), such as increased total ceramides (Extended Data Fig. 9c), PUFA/non-PUFA TG ratio (Extended Data Fig. 9d) and a number of SMs and TGs (Extended Data Fig. 9g–i).

**Fig. 7:**
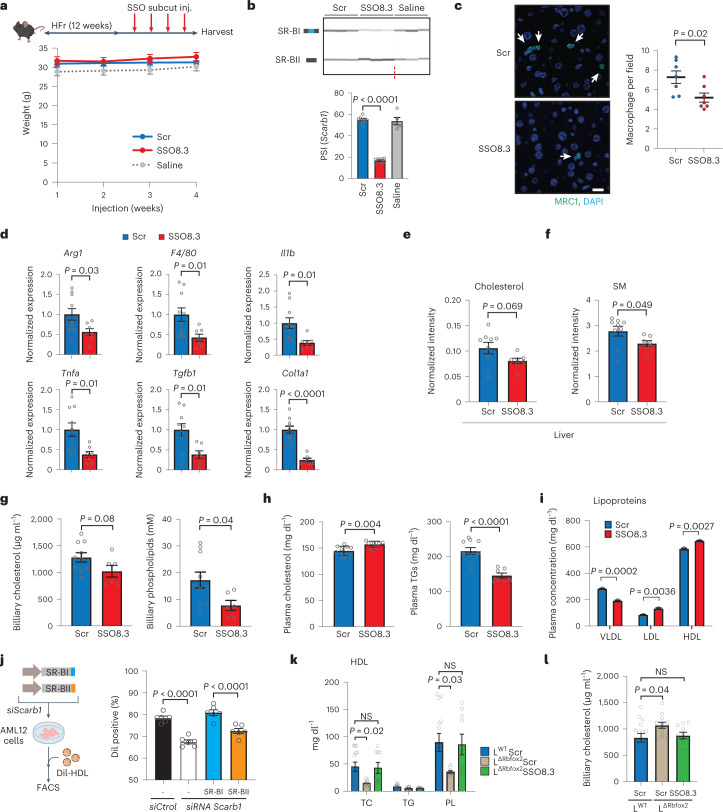
*Scarb1* mediates lipidomic changes associated with RBFOX2 deficiency in hepatocytes and can be regulated with splice-switching oligos. **a**, Male mice fed a HFr diet were subcutaneously injected with SSO8.3, scramble (Scr) or saline during four consecutive weeks (top). No significant effect of injections on body weight was detected (bottom). **b**, Semiquantitative PCR analysis of *Scarb1* exon 12 inclusion in liver (top) and quantification of overall AS caused represented as PSI (bottom). **c**, Immunohistochemistry showing decreased macrophage (anti-MRC1) infiltration in the liver of SSO8.3-treated mice. Nuclei were stained with DAPI. Scale bar 10 µm. **d**, qPCR analysis in the liver of SSO8.3-treated mice (*n* = 7–9). **e**,**f**, LC–MS lipidomic analysis showing total levels of free cholesterol (**e**) and sphingomyelin (SM) (**f**) normalized to the tissue weight in mice fed a HFr diet on SSO8.3 injection. **g**, Quantification of cholesterol and phospholipids in the bile of SSO8.3- and Scr-treated mice. **h**, Blood analysis of SSO8.3- versus Scr-treated mice showing total cholesterol and TGs. **i**, Analysis of blood VLDL, LDL and HDL lipoprotein composition. Samples were pooled in three replicates. **j**, Cartoon depicting the analysis of Dil-HDL uptake in AML12 hepatocytes expressing codon-optimized SR-BI or SR-BII after targeted inactivation of endogenous *Scarb1* with a specific siRNA or scramble control (left). Uptake as quantified as Dil-positive cells after 4 h of incubation with 0.1 μg ml^−1^ Dil-HDL (*n* = 6). **k**, Quantification of main lipid species in HDL lipoproteins purified from L^Δ*Rbfox2*^ versus L^WT^ male mice fed a HFr diet and treated with SSO8.3 or Scr control. **l**, Cholesterol levels quantified in the bile of L^Δ*Rbfox2*^ versus L^WT^ mice treated with SSO8.3 or Scr control as indicated (*n* = 7–10). Results are represented as mean ± s.e.m. Statistical significance was determined by two-tailed unpaired *t*-test (**c**–**i**) or one-way ANOVA and Dunnett’s multiple comparisons test (**b**, **j**–**l**) of biologically independent samples. Source data

Increased intrahepatic levels of cholesterol, ceramides, sphingomyelins and other lipotoxic species have been implicated in the pathogenesis of obesity-induced steatohepatitis^13,38^. While *SCARB1* is a complex therapeutic target, the effect of SSO8.3 in decreasing the amounts of some of these lipid species in hepatocytes indicates that strategies aimed to promote the SR-BII isoform could contribute to alleviating obesity-induced inflammation in vivo. To test this, we injected SSO8.3, a scrambled control SSO (Scr) or saline into mice fed an obesogenic HFr diet (Fig. 7a, top). SSO8.3 showed potent activity in antagonizing *Scarb1* exon 12 inclusion in vivo in the liver (Fig. 7b and Extended Data Fig. 9j). SSO8.3 specificity was confirmed by evaluating expression of the top three potential off-target genes by qPCR (Extended Data Fig. 9k–m). The RNA injections were not associated with toxic side-effects (either body weight loss (Fig. 7a, bottom) or increase in transaminases (Extended Data Fig. 9n–o)). Immunohistochemistry of liver sections showed decreased macrophage infiltration in liver from mice injected with SSO8.3 (Fig. 7c). qPCR showed that expression of inflammatory (*Arg1*, *F4/80*, and *Tnfa*) and fibrotic (*Tgfb1* and *Col1a1*) markers was significantly down-regulated in mice treated with SSO8.3 (Fig. 7d). Consistent with a decreased lipid load in hepatocytes (Fig. 7e,f), SSO8.3-treated mice showed a decreased liver/total body weight ratio (Extended Data Fig. 9p) and decreased cholesterol and phospholipid secretion into the bile (Fig. 7g) suggesting decreased reverse cholesterol transport into the liver.

**Extended Data Fig. 10:**
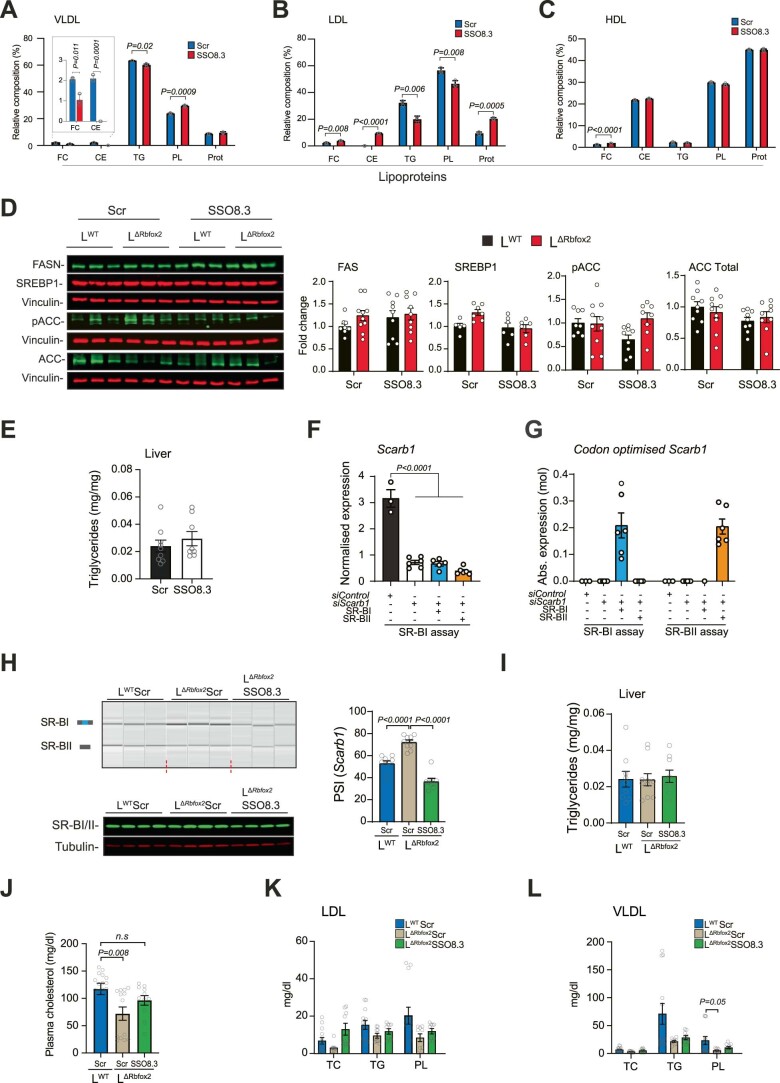
*In vivo* treatment with SSO8.3 promotes lipoprotein remodelling. A.- Quantification of major lipid species in purified VLDL, B.- LDL and C.- HDL lipoproteins from mice fed a HFr diet and treated with SSO8.3 or Scr control. Samples were pooled in three replicates. Results are represented as mean ± SEM; Statistical significance was determined by unpaired *t*-test corrected for multiple comparisons using the Holm-Sidak method. D.- Representative western blot analysis of lipogenic proteins in liver of L^WT^ and L^Δ*Rbfox2*^ mice treated with SSO8.3 or Scr (n = 9-10). E.- Quantification of liver triglyceride content normalised to liver weight in mice injected with SSO8.3 or Scr control (n = 8-9). F.- RT-qPCR expression analysis of endogenous *Scarb1* gene knockdown in AML12 hepatocytes treated with siRNA to *Scarb1* or Scr control (n = 3-6). Results are represented as mean ± SEM. Statistical significance was determined by one-way ANOVA and Dunnett’s multiple comparisons test of biologically independent samples. G.- Absolute RT-qPCR expression analysis of codon-optimised *Scarb1* isoforms in AML12 cells (n = 3-6). H.- Capillary electrophoresis and quantification of percentage splice in (PSI) for *Scarb1* (exon 12) in L^WT^ and L^Δ*Rbfox2*^ liver upon treatment with SSO8.3 or Scr control (top) and western-blot analysis of protein levels (bottom) (n = 8-9). I.- Quantification of liver triglyceride content normalised to liver weight in L^WT^ and L^Δ*Rbfox2*^ mice upon treatment with SSO8.3 or Scr control (n = 9-10). J.- Total blood cholesterol level in L^Δ*Rbfox2*^ vs. L^WT^ mice fed a HFr diet and treated with SSO8.3 or Scr control as indicated (n = 11-15). K.- Quantification of major lipid species in purified LDL and (L) VLDL lipoproteins (n = 9-13). Results in H-L are represented as mean ± s.e.m. Statistical significance was determined by one-way ANOVA and Dunnett’s multiple comparisons test of biologically independent samples. Source data

Genetically modified mouse models have shown that complete^39^ or liver-specific^40^ inactivation of *Scarb1* is associated with increased VLDL, LDL and HDL levels, and increased atherosclerosis, while over-expression of SR-BI has the opposite effect^28^. SSO8.3 treatment caused a mild but significant increase in total cholesterol levels and a decrease in blood TGs (Fig. 7h). These results indicate that by increasing SR-BII isoform expression, SSO8.3 treatment leads to specific effects on VLDL and HDL lipoproteins. Consistent with this hypothesis, lipoprotein analysis showed a substantial decrease in VLDL lipoproteins and increased HDL and LDL levels (Fig. 7i). To confirm these findings, lipid species content was quantified in isolated lipoproteins, revealing the main alterations in lipoprotein composition, including a drop in the cholesterol content of VLDL, whereas that of HDL and LDL was increased on SSO8.3 treatment (Extended Data Fig. 10a–c). SSO8.3 treatment was not associated with changes in lipogenic genes (Extended Data Fig. 10d) or overall TG content in the liver (Extended Data Fig. 10e). For these reasons, while we cannot exclude a contribution from decreased VLDL secretion, the decreased total VLDL and LDL TG blood level suggests that SSO8.3 accelerates VLDL lipolysis and remnants formation. Confirmation of this mechanism will require further investigation.

Thus, RBFOX2 coordinates an AS network in the liver that promotes specific changes in lipid metabolism and collectively regulates the homeostasis of lipid species including cholesterol, sphingomyelins and phospholipids. Additionally, the SR-BI/II splice switch can be targeted therapeutically to decrease liver inflammation and modify lipid distribution.

### *Scarb1* AS mediates the effect of RBFOX2 in cholesterol metabolism

RBFOX2 deficiency is not associated with changes in cholesterol biosynthesis nor lipogenesis (Extended Data Figs. 5c and 10d). Moreover, our data support that increased SR-BI-mediated lipid uptake contributes to increased accumulation of cholesterol and other lipids on RBFOX2 deficiency. We proposed that this mechanism could also underlie the changes in blood cholesterol associated with RBFOX2 deficiency, through increased reverse cholesterol uptake and excretion into the bile. To test this idea, we repressed endogenous *Scarb1* expression using small-interfering RNA (siRNA) in AML12 hepatocytes that simultaneously expressed either codon-optimized SR-BI or SR-BII (Fig. 7j and Extended Data Fig. 10f). Comparable expression levels of SR-BI and SR-BII were confirmed by absolute qPCR quantification (Extended Data Fig. 10g). Fluorescence activated cell sorting (FACS) analysis showed that expression of the SR-BI isoform is associated with increased lipid uptake from 1,1′-dioctadecyl-3,3,3′,3′-tetramethylindocarbocyanine perchlorate (Dil)-HDL lipoproteins (Fig. 7j).

To investigate how SR-BI/II isoforms contribute to the role of RBFOX2 in vivo, we treated L^WT^ and L^Δ*Rbfox2*^ mice with Scr control or SSO8.3 to antagonize the SR-BI isoform in L^Δ*Rbfox2*^ mice fed a HFr diet. SSO8.3 efficiently reverted the SR-BI to SR-BII ratio in L^Δ*Rbfox2*^ mice liver (Extended Data Fig. 10h). Blood lipoproteins were isolated and lipid composition was quantified. Cholesterol and phospholipid content in HDL was decreased in L^Δ*Rbfox2*^ mice and these differences were abolished by reverting the SR-BI to SR-BII ratio, confirming the contribution of this isoform switch to RBFOX2 role in the liver (Fig. 7k). Cholesterol in the bile was increased on RBFOX2 inactivation in the liver (Fig. 7l), while transient RBFOX2 over-expression causes the opposite effect (Fig. 5g). Switching *Scarb1* isoform expression through SSO8.3 treatment reverted both cholesterol excretion in the bile (Fig. 7l) and total blood cholesterol levels (Extended Data Fig. 10j), further confirming that SR-BI/II-mediated reverse cholesterol transport underlies the role of RBFOX2 in cholesterol metabolism. Purification and analysis of blood LDL and VLDL showed that RBFOX2 deficiency was associated with changes in cholesterol that did not reach statistical significance, suggesting an important role for RBFOX2 in cholesterol HDL uptake (Extended Data Fig. 10k,l). Collectively, these results uncover a role for RBFOX2 in the control of lipid metabolism under a lipogenic diet and show that the SR-BI/SR-BII isoform switch is critical for this mechanism by regulating HDL lipoprotein homeostasis.

## Discussion

In the liver, pre-mRNA AS has been regarded as a housekeeping mechanism involved in tuning the transcriptome to maintain cellular identity. While several splicing factors play a role in this regulation^41–45^, the contribution of specific AS networks (splicing factors and downstream isoforms) to fluctuating metabolic demands remains to be characterized.

Here, we describe a role for RBFOX2 in regulating genes involved in lipid metabolism in the liver. This AS network is modulated by an obesogenic diet, and RBFOX2 is critical for this regulation. In mammals, the *Rbfox* family includes three paralogues: *Rbfox1*, *Rbfox2* and *Rbfox3*. *Rbfox1* is expressed in neurons, heart and muscle; *Rbfox3* expression is restricted to neurons and *Rbfox2* has a broader expression profile^46^. We have found that *Rbfox2* is mainly expressed in hepatocytes in the liver, and under a lipogenic diet, ablation of *Rbfox2* gene in hepatocytes leads to a cholesterol decrease in the blood and an increase in intrahepatic content of cholesterol, bile acids and other lipids, uncovering a role for RBFOX2 in controlling lipid distribution.

Increased circulating and intrahepatic cholesterol levels contribute to MAFLD and promote coronary artery disease^14,15^. Liver cholesterol overload also contributes to progression of liver damage and inflammation^13,38,47^. Cholesterol levels are tightly regulated to ensure a constant supply to tissues, while preventing the detrimental effects of excessive accumulation. The RBFOX2 AS network illustrates the complexity of cholesterol homeostasis in health and disease.

We demonstrate that RBFOX2-mediated regulation of splicing variants in *Scarb1*, *Pla2g6*, *Numb*, *Sec31a* or *Osbpl9* transcripts is conserved in humans and has roles in controlling lipid composition. While the coordinated activity of this splicing network underlies the effect of RBFOX2 in lipid metabolism, individual components can be targeted with SSOs to trigger specific changes in hepatocyte lipid content. Two different canonical receptors, SR-BI and SR-BII, are generated through the inclusion/skipping of exon 12 of the *Scarb1* gene. SR-BI has increased activity in HDL binding and promotes the selective import of cholesteryl esters^37,48^. However, the regulation and the biological significance of this splicing event had not been described. HDL-cholesterol levels in plasma are inversely correlated with atherosclerosis risk in humans and in some murine models^49^. For this reason, SR-BI is considered a therapeutic target for modifying lipid metabolism by boosting HDL-C levels. However, human genetic studies^50^ together with gain-^28^ and loss-of-function^39^ mouse models have shown that SR-BI activity is atheroprotective, demonstrating that HDL-cholesterol flux is more important than the steady state levels. We find that by promoting SR-BII expression, RBFOX2 prevents reverse cholesterol flux from HDL lipoproteins, a mechanism that ensures appropriate distribution and prevents excessive cholesterol loss. Consistently, RBFOX2 inactivation is associated with decreased total and HDL cholesterol in the blood, and increased cholesterol in the liver and consequent excretion in the bile under a lipogenic diet. Moreover, by promoting SR-BII expression, the SSO SSO8.3 substantially reduces the accumulation of lipotoxic species such as cholesterol and sphingomyelins, and this effect is associated with decreased expression of inflammatory markers in the liver.

Treatment with SSO8.3 to promote SR-BII isoform expression in vivo is associated with a substantial reduction in plasma VLDL and TGs, showing that rather than a loss or a gain of function, SR-BII isoform expression promotes an anti-atherogenic lipoprotein profile, potentially by accelerating VLDL catabolism. While our results establish that the RBFOX2-SR-BI/II axis plays a key role in controlling cholesterol homeostasis, the contribution of the SR-BI/II splice switch to TG homeostasis seems more complex as RBFOX2 inactivation is not associated with changes in total circulating TGs. Similarly, the changes in cholesterol levels associated with RBFOX2 deficiency are mainly detected under a HFr diet, suggesting a role of this AS network in controlling cholesterol homeostasis under lipogenic conditions.

RNA-based drugs such as inclisiran^8^ and Mipomersen^7^ significantly reduce cholesterolemia by targeting PCSK9 and APOB mRNAs, respectively. Recent improvements in the design and pharmacokinetics of RNA-based oligonucleotides should enable the development of isoform-specific therapeutics for common metabolic pathologies. However, this avenue is underexplored due to the limited characterization of the key isoforms maintaining health or promoting disease. Our work provides a proof-of-principle for the potential of RNA therapeutics targeting individual isoforms in the liver.

## Methods

### Mice

C57BL6/J (stock number 000664), *Rbfox2*^*loxP/loxP*^ (stock number 014090)^51^ and Albumin-cre (stock number 003574)^52^ were obtained from the Jackson Laboratory. Eight-week-old mice were randomly assigned to experimental groups and fed a CD, an HFD (60% kcal from fat, Bioserve) or a HFr diet containing 30% (w/v) fructose, administered in the drinking water for 16–22 weeks. Male mice were used, unless otherwise indicated in the text. Mice were housed in pathogen-free barrier facilities under a 12 h light/dark cycle at 22 °C with controlled humidity and free access to food and water. The presence of the Cre recombinase and *Rbfox2* LoxP sites was determined by PCR analysis of genomic DNA and the following primers: CreF1>TTACTGACCGTACACCAAATTTGCCTGC and CreR1>CCTGGCAGCGATCGCTATTTTCCATGAGTG, Rbfox2F1> AACAAGAAAGGCCTCACTTCAG and Rbfox2R1>GGTGTTCTCTGACTTATACATGCAC. All in vivo work was approved by the animal welfare and ethical review board at Imperial College London and in accordance with the United Kingdom Animals (Scientific Procedures) Act (1986).

### Adeno viruses and AAVs

The adenoviral vector driving mouse RBFOX2 expression (Ad-m-RBM9) and pAd-GFP were obtained from Vector Biolabs. These viruses were purified by using the AdEasy virus purification kit (Agilent technologies). Then 8 × 10^9^ GC were intravenously injected in mice fed a HFr diet and mice were harvested 7 days postinjection.

Codon-optimized RBFOX2 lacking the RRM was obtained by gene block synthesis (IDT) and cloned in to the AAV-CBA-GFP vector. AAV2/8 were produced and purified by iodixanol gradients and 5 × 10^11^ GC were intravenously injected in mice fed a HFr diet. Mice were harvested 12 weeks postinjection.

### Tissue and blood harvesting

Biopsies were flash frozen in liquid nitrogen and kept at −80 °C. Sections for histology were fixed in 10% formalin and subsequently embedded in paraffin, or immersed in optical cutting temperature compound and isopentane, for cryosections. Paraffin sections were stained with haematoxylin & eosin, or used for immunohistochemistry analysis of macrophage infiltration with anti-MRC1 antibody (ab64693) and 4,6-diamidino-2-phenylindole (DAPI) (Sigma, D9542) for nuclear staining. Plasma was obtained in EDTA tubes by centrifugation at 5,000*g* for 10 min at 4 °C and analysed by St Mary’s Hospital pathology department unless otherwise indicated.

### Quantitative proteomics (TMT–MS)

Flash frozen livers were lysed using a homogenizer with SDS lysis buffer (2.5% SDS, 50 mM HEPES pH 8.5, 150 mM NaCl, 1× EDTA-free protease inhibitor cocktail (Roche), 1× PhosSTOP phosphatase inhibitor cocktail (Roche)). Lysates were clarified by centrifugation at 15,000*g* for 15 min and protein concentration was measured by Pierce BCA assay (Thermo Scientific). Then 20 mg of protein was reduced with 5 mM TCEP for 30 min, then alkylated with 14 mM iodoacetamide for 30 min and finally quenched with 10 mM DTT for 15 min. All reactions were performed at room temperature. Proteins were chloroform-methanol precipitated and the pellet resuspended in 8 M urea, 50 mM EPPS pH 8.5. To help with the resuspension, protein precipitates were passed ten times through a 22G needle and protein concentration was measured again. Before protein digestion, 5 mg of protein was collected, and urea concentration diluted to 1 M with 50 mM EPPS pH 8.5. Then, LysC was added at 1:100 (LysC:protein) and digested for 12 h at room temperature. Samples were further digested for 5 h at 37 °C with trypsin at 1:100 (trypsin:protein). To stop the digestion 0.4% TFA (pH < 2) was added to the samples. Digested samples were clarified by centrifugation at 15,000*g* for 10 min. Peptide concentration was measured using a quantitative colorimetric peptide assay (Thermo Scientific). Next, 25 μg of peptides were desalted using 10 mg SOLA HRP SPE Cartridges (Thermo scientific). To allow the comparison of both TMT, two bridge channels were prepared and processed in parallel. For that, 1.39 μg of each sample was added for to each bridge channel. Then, dried peptides from all 20 samples were resuspended in 200 mM EPPS pH 8.5 and labelled with TMT-10plex following the protocol described in^53^. After labelling, both bridge channels were combined and split again to ensure homogeneity. Finally, samples were mixed in equal amounts. After combining, both TMT were desalted using the tC18 SepPak solid-phase extraction cartridges (Waters) and dried in the SpeedVac. Next, desalted peptides were resuspended in 5% ACN, 10 mM NH_4_HCO_3_ pH 8. Both TMT were fractionated with basic pH reversed-phase chromatography using a high-performance liquid chromatography (HPLC) set-up equipped with a 3.5 µm Zorbax 300 Extended-C18 column (Agilent). 96 fractions were collected and combined into 24. Of these, 12 were desalted following the C18 Stop and Go Extraction Tip (STAGE-Tip)^54^ and dried down in the SpeedVac. Finally, samples were resuspended in 3% ACN, 1% FA and run in an Orbitrap Fusion running in MS3 mode^55^ as described previously^53^. RAW data were converted to mzXML format using a modified version of RawFileReader and searched using the search engine Comet^56^ against a mouse target-decoy protein database (Uniprot, 11 June 2019) that included the most common contaminants. Precursor ion tolerance was set at 20 ppm and product ion tolerance at 1 Da. Cysteine carbamidomethylation (+57.0215 Da) and TMT tag (+229.1629 Da) on lysine residues and peptide N termini were set up as static modifications. Up to two variable methionine oxidations (+15.9949 Da) and two miss cleavages were allowed in the searches. Peptide-spectrum matches were adjusted to a 1% FDR with a linear discriminant analysis^57^ and proteins were further collapsed to a final protein-level FDR of 1%. TMT quantitative values we obtained from MS3 scans. Only those with a signal-to-noise >100 and an isolation specificity >0.7 were used for quantification. Each TMT was normalized to the total signal in each column. To allow the comparison of both TMT, those proteins quantified in both TMT, data were normalized using the bridge channels present in each TMT. Quantifications included in Supplementary Table 1 are represented as relative abundances. Newly generated proteomic datasets are publicly available as described below.

### iPSC-derived human hepatocytes

Human iPSCs CGT-RCiB-10 (Cell & Gene Therapy Catapult) were maintained on Vitronectin XF (STEMCELL Technologies) coated Corning Costar TC‐treated six‐well plates (Sigma Aldrich) in Essential 8 Medium (Thermo Fisher Scientific) and passaged every 4 days using Gentle Cell Dissociation Reagent (STEMCELL Technologies).

Hepatocyte differentiation was carried out as previously described^58,59^. Silencing of human *RBFOX2* was performed by transfecting 100 nM smart pool to *RBFOX2* or a mock control (Horizon) with RNAimax reagent (Invitrogen) in OptiMEM (Invitrogen).

### AML12 hepatocytes and Dil-HDL uptake

AML12 originally obtained from ATCC (ATCC CRL-2254) were cultured as previously described^60^. Silencing of *Scarb1* was performed by transfecting 100 nM smart pool to *Scarb1* or a mock control (Horizon) with RNAimax reagent (Invitrogen) in OptiMEM (Invitrogen). Expression of SR-BI and SR-BII was obtained by cloning codon-optimized SR-BI and SR-BII (generated by gblock synthesis at IDT) into pLV (PGK)-GFP Neo vector. Third generation lentiviruses were generated in human embryonic kidney-293T cells (ATCC CRL-1573) and purified by high-speed centrifugation. Viruses were resuspended in media supplemented with polybrene and added to the cells. When indicated, cells were incubated with 100 ng ml^−1^ Dil-HDL for 4 h and lipid uptake was quantified by FACS using a FACSAria III cell sorter system (BD Biosciences).

### RNA isolation

Cells or tissues were homogenized in TRIzol (Thermo Fisher Scientific) and RNA was extracted following the manufacturer’s instructions. For RNA-seq, after homogenization with TRIzol, RNA was extracted with a RNeasy kit column (Qiagen) following the manufacturer’s instructions, including DNase I treatment.

### RNA-seq sequencing and analysis

RNA was quality controlled with a 2100 BioAnalyser (Agilent). Poly(A) enrichment of samples with RNA integrity number > 8, was performed and libraries were prepared using the NEBNext Ultra II RNA Library Prep Kit for Illumina and multiplexed using NEBNext Multiplex Oligos for Illumina (NEB, E7760S and E7335S). Sequencing was carried out with 100 basepair paired end reads with HiSeq 2500 (Illumina). Newly generated RNA-seq datasets are publicly available as described below. Previously published datasets of mice fed a HFr diet were obtained from GSE123896 (ref. ^16^). Data were processed using RTA v.1.18.54, with default filter and quality settings. The reads were demultiplexed with CASAVA v.2.17. Reads were aligned to Ensembl mouse genome (GRCm38) using Tophat2 (v.2.0.11)^61^ with the argument ‘–library-type fr-firststrand’. Reference sequence assembly and transcript annotation were obtained from Illumina iGenomes (https://support.illumina.com/sequencing/sequencing_software/igenome.html). Gene-based read counts were obtained using featureCounts function from Rsubread Bioconductor package^62^. Normalization and differential expression analysis were performed using DEseq2 (ref. ^63^) or edgeR-voom-limma^64–67^ bioconductor packages. AS was primarily analysed with rMATs^68^. Differentially spliced sites were kept for data visualization if they passed the following threshold: *P* < 0.05; FDR < 0.1 and absolute(IncLevelDifference) >0.1 or <-0.1. Gene ontology analysis was carried out by using GOseq Bioconductor package^69^. A list of differentially expressed genes with adjusted *P* value of less than or equal to 0.05 were selected as input for the ingenuity pathway analysis (http://www.ingenuity.com/index.html). No cut-off was applied for fold change of differential expression. Enrichment of binding motifs for different splicing factors were tested using the binomTest function from the edgeR bioconductor package^70^.

### Lipidomics analysis

Tissue was pulverized using a cyroPREP Dry Pulverizer (Covaris). Adapted from Folch and colleagues^71^. Approximately 30 mg of frozen liver powder was weighed into a weighed Eppendorf. Tissue was homogenized in a TissueLyzer (20 Hz, 3–5 min × 2) using a stainless steel ball and 1 ml chloroform:methanol (2:1). The stainless steel ball was removed and 400 μl HPLC-grade water was added, samples were vortexed for 20 s and centrifuged for 15 min, 13,200*g* at room temperature. Both the organic and the aqueous layers were removed. The protein pellet was re-extracted in 500 μl of 2:1 chloroform:methanol and 200 μl of HPLC-grade water, samples were vortexed and centrifuged, and the respective fractions were combined. For hepatocyte analysis, following trypsinization cells were centriguged at 180*g* for 5 min at room temperature. Lipids were extracted from dried pellet following the same procedure. Lipid profiling was performed by LC–high-resolution MS using a Vanquish Flex Binary UHPLC system (Thermo Scientific) coupled to a benchtop hybrid quadrupole Orbitrap Q-Exactive mass spectrometer (Thermo Scientific). Chromatographic separation was achieved using an Acquity UPLC BEH C18 column (Waters, 50 × 2.1 mm, 1.7 µm) held at a temperature of 55 °C and flow rate of 0.5 ml min^−1^. For the positive ion mode, the mobile phase was composed of 60:40 (v/v) acetonitrile:water plus 10 mM ammonium formate (solvent A) and 90:10 (v/v) isopropanol:acetonitrile plus 10 mM ammonium formate (solvent B). For the negative ion mode, the mobile phase was composed of 60:40 (v/v) acetonitrile:water plus 10 mM ammonium acetate (solvent A) and 90:10 (v/v) isopropanol:acetonitrile plus 10 mM ammonium acetate (solvent B). The gradient elution program was performed for both ion modes according to the Supplementary Table 4, yielding a total run time of 10 min per sample. The injection volume for the positive and negative ion mode was 5 and 10 µl, respectively. The ionization was performed using a heated electrospray ionization source and the parameters for the positive or negative mode are as follows: capillary voltage 3.5 or −2.5 kV, heater temperature 438 °C, capillary temperature 320 °C, S-lens radio frequency level 50, sheath, auxiliary and sweep gas flow rate are 53, 14 and 1 unit, respectively. The mass accuracy was calibrated before sample analysis for both ion modes. High-resolution mass spectrometric (70,000 at *m/z* 200) data were acquired in profile mode using the full scan setting (*m/z* 200–2,000). Automatic gain control was set to 1 × 10^6^ and maximum MS1 injection time at 200 ms. Lipidomics data acquisition was performed with Xcalibur software (v.4.1). Peak picking was performed using XCMS^72^, and features normalized to isotopically labelled internal standard and dry tissue weight. For in vitro anlysis, intensities were normalized to internal standard and total ion count unless otherwise indicated. Lipid identification was performed by accurate mass using an in‐house database.

Bile acid analysis was carried out using LC–MS/MS in an Acquity I-Class binary UPLC system (Waters) coupled to a triple quadrupole Xevo TQ-XS mass spectrometer as previously described (https://www.waters.com/webassets/cms/library/docs/720006261en.pdf) (Waters). Chromatographic separation was performed on a CORTECS T3 column (Waters, 30 × 2.1 mm, 2.7 µm) held at a temperature of 60 °C and flow rate of 1.3 ml min^−1^. Mobile phase consisted of 0.2 mM ammonium formate plus 0.01% (v/v) formic acid (solvent A) and 50:50 (v/v) isopropanol:acetonitrile plus 0.01% (v/v) formic acid and 0.2 mM ammonium formate. The elution gradient program started with 20% of B holding for 0.1 min and ramping up to 55% of B over 0.7 min, followed by 0.9 min of column washing at 98% of B. The column was re-equilibrated to initial conditions, yielding a total run time of 1.71 min per sample. The injection volume was 10 µl. Data were acquired using multiple reaction monitoring in the negative ion mode according to Supplementary Table 5. The source parameters were as follows: −2.0 kV capillary voltage, 60 V cone voltage, desolvation temperature 600 °C and cone and desolvation gas flow rates were 150 and 1,000 l h^−1^, respectively. Data were acquired by MassLynx software (v.4.2) and processed with TargetLynx XS (Waters).

### Quantification of liver TGs

Liver (50–200 mg) was incubated overnight at 50 °C added in 350 μl of ethanolic KOH (2 ethanol (100%):1 KOH (30%)). Following incubation, samples were vortexed and 650 μl of ethanol (50%) was added followed by centrifugation at full speed for 5 min. Then 900 μl of the of the supernatant was mixed with 300 μl of ethanol (50%) and 200 μl of the samples were mixed with 215 μl of 1 M MgCl_2_, and incubated on ice for 10 min. Subsequently, samples were centrifuged at full speed for 5 min and 10 μl of the supernatant was assayed for glycerol content using free glycerol reagent (Sigma).

### Protein analysis

Tissue was homogenized in Triton Lysis Buffer (12.5 mM HEPES pH 7.4, 50 mM NaCl, 500 μM EDTA, 5% glycerol, 0.5% Triton X-100, 50 mM sodium vanadate, 50 mM phenylmethyl sulfonyl fluoride, 5 mM aprotinin, 5 mM, Leupeptin) using a TissueLyser II Homogenizer (Qiagen) before undergoing centrifugation at 10,000*g* for 10 min at 4 °C. The supernatant was transferred to a new tube and protein quantified with Pierce BCA Protein Assay Kit (Thermo Fisher Scientific) and analysed by western blot by incubating with anti-RBFOX2 (Bethyl Laboratories), anti-PLA2G6 (Santa Cruz), anti-SREBP1 (Pharmigen), anti-vinculin (Sigma), anti-APOB (Proteintech), anti-ABCA1 (Invitrogen), anti-ACC, anti-pS79 ACC, anti-FASN (Cell Signaling) and anti-Tubulin (Santa Cruz) primary antibodies diluted 1:1,000 in blocking buffer (LI-COR) and secondary 680/800 nm antibodies (LI-COR) diluted 1:5,000. Membranes were imaged with an Odyssey infra-red scanner (LI-COR).

### Glucose tolerance tests

For glucose tolerance tests animals are fasted for 16 h and intraperitoneally injected with 1 g of glucose per kg. Blood glucose was measured using Contour XT glucometers (Roche).

### Lipoprotein fractionation and characterization

The main lipoprotein fractions, namely very low-density lipoprotein (VLDL, *d* <1.019 g ml^−1^), low-density lipoprotein (LDL, *d* 1.019–1.063 g ml^−1^) and high-density lipoprotein (HDL, *d* 1.063–1.21 g ml^−1^), were successively isolated from plasma by sequential ultracentrifugation at 513,000*g* at 15 °C using a Beckman Optima Max-TL centrifuge following periods of centrifugation of 1 h 30, 3 h 30 min and 5 h 30 min, respectively. After isolation, lipoprotein fractions were analysed for their lipid and protein content with a calibrated AutoAnalyzer (Konelab 20) by using commercial kits. Total cholesterol (TC), free cholesterol (FC) and phospholipids were measured using reagents from Diasys. Cholesteryl ester mass was calculated as (TC − FC) × 1.67 and thus represents the sum of the esterified cholesterol and fatty acid moieties. TGs were quantified with a commercial kit (Thermo Electron). Bicinchoninic acid assay reagent (Pierce, Thermo Fisher Scientific) was used for protein quantification. Lipoprotein mass was calculated as the sum of the mass of the individual lipid and protein components for each lipoprotein fraction.

### Primary hepatocytes

Livers were perfused using liver perfusion buffer (HBSS KCl 0.4 g l^−1^, glucose 1 g l^−1^, NaHCO_3_ 2.1 g l^−1^, EDTA 0.2 g l^−1^) and then digested using liver digest buffer (DMEM-GlutaMAX 1 g l^−1^ glucose, HEPES 15 mM pH 7.4, penicillin/streptomycin 1%, 5 mg per mouse Collagenase IV (C5138 Sigma)). After excision, livers were placed on ice in plating media (M199, foetal bovine serium 10%, penicillin/streptomycin 1%, sodium pyruvate 1%, l-glutamine 1%, 1 nm insulin, 1 mM dexamethasone, 2 mg ml^−1^ bovine serum albumin (BSA)). Tissue was homogenized using forceps and then filtered in plating media. Cells were then washed twice in plating media and then subjected to a 1:3 Percol Gradient (Sigma Aldrich). Cells were plated on collagen coated plates (Thermo Fisher Scientific) in plating media. Media was changed after 3 h to maintenance media (DMEM 4.5 g of glucose per l, penicillin/streptomycin 1%, l-glutamine 1%, 100 nM dexamethasone, 2 mg ml^−1^ BSA) for 12 h, and hepatocytes were treated as indicated.

### Generation of cell lines expressing pGIPZ lentiviral short hairpin RNA (shRNA)

Lentiviral shRNA (sh1-Foxa1, *v2lmm14620*; sh4-Foxa2, *v2lmm71498)* clones were recovered from the pGIPZ library following the manufacturer’s protocol (Thermo Fisher) and used to obtain lentiviruses and produce stable Hepa1-6 cells originally obtained from ATCC (CRL-1830).

### Antisense oligonucleotides

RNA SSOs were synthetized with 2′O-ME modifications and phosphorothioate backbone (Eurogentec). A complete list of SSO sequences is provided in Supplementary Table 6. Then, 100 nM SSO were transfected by incubating cells with Lipofectamine2000 in OptiMEM (Thermo Fisher scientific). For in vivo studies, 40 mg kg^−1^ per week of oligonucleotides or saline were weekly injected subcutaneously over four consecutive weeks.

### CAGE-seq and ChIP–seq data processing

Mapped CAGE-supported transcriptional start sites from the FANTOM5 project^73–75^ were imported to R (http://www.R-project.org/) as CAGE-supported transcriptional start site tables. Replicate samples were merged and normalized using the standard workflow within the CAGEr package^76^. Processed bigwig files corresponding to human adult liver ChIP–seq signal *P* value were obtained from the ENCODE portal^77^: FOXA1 (ENCFF058DKS)^78^, FOXA2 (ENCFF902TMK)^78^, K3K4me3 (ENCFF610REU)^79^ and H3K27ac (ENCFF012XAP)^79^. Mouse liver FOXA1 ChIP–seq data (GSE106379) was retrieved from ref. ^80^.

### PCR with reverse transcription (RT–PCR) analysis

RNA was reverse transcribed using High-Capacity complementary DNA Reverse Transcription Kit (Thermo Fisher Scientific). Taqman Gene Expression Assays (Thermo Fisher Scientific) with probes from the Roche Universal Probe Library or Fast SYBR-Green Mix (Thermo Fisher Scientific) were used for quantification on QuantStudio 7 Flex Real-Time PCR system (Thermo Fisher Scientific). All data were analysed using a relative standard curve or the delta CT method. Ribosomal 18S RNA was used to normalize samples in all cases. AS analysis was carried out using primers designed for detecting more than one mRNA isoform. Targets were amplified by PCR and were analysed by capillary electrophoresis by using the QIAxcel Advanced System (Qiagen). AS was calculated as percentage spliced in (PSI) of a specific splicing event across samples (PSI = (long isoform)/(long isoform + short isoform) × 100). Probes and primers used in this analysis are described in Supplementary Table 3.

### Ultra-violet eiCLIP

A revised version of the previously described non-isotopic individual-nucleotide-resolution UV iCLIP workflow^81^ was carried out with new modifications to enhance speed and efficiency. Specifically, a shortened Cy5.5 labelled adaptor was incorporated (/5Phos/A[XXXXXX]NNNAGATCGGAAGAGCACACG/3Cy55Sp/), high concentration T4 RNA ligase (New England Biolabs) was used to enhance adaptor ligation, the RecJf exonuclease (New England Biolabs) was used to remove un-ligated adaptor before SDS–PAGE, SDS–PAGE was visualized in the 700 nm channel, reverse transcription was carried out with a biotinylated primer homologous to the adaptor (/5BiotinTEG/CGTGTGCTCTTCCGA), un-incorporated RT-primer was removed by exonuclease III (New England Biolabs) after annealing to the reverse complement, cDNA was captured by MyOne streptavidin C1 magnetic beads (Thermo Fisher Scientific), bead bound cDNA was ligated to a 3′ adaptor (/5Phos/ANNNNNNNAGATCGGAAGAGCGTCGTG/3ddC/) instead of carrying out the previous used intramolecular ligation and cDNA was eluted from the streptavidin beads using nuclease and cation free water at high temperature. A 5% size-matched input was prepared by capturing the cellular proteome on SeraMag carboxylic beads (Sigma Aldrich) and proceeding through the eiCLIP protocol in parallel with RBFOX2 immunoprecipitated complexes. RBFOX2 eiCLIP was performed using 1 μg μl^−1^ RBM9 Antibody (A300-A864A, Bethyl Laboratories) using isolated primary hepatocytes: total protein was quantified at 4 μg μl^−1^ and antibody was added to 8 μg ml^−1^. Samples from three independent hepatocyte cultures each obtained from two mice were sequenced with paired end reads using a MiSeq system (Illumina).

### Mapping and identification of crosslink clusters from eCLIP and eiCLIP experiments

For mapping eCLIP and eiCLIP-RBFOX2 sequencing data, we used GENCODE assembly annotation version ‘GRCm38.VM20’ for mouse and ‘GRCh38.p7’ for human samples. For the eCLIP samples a double adaptor removal was used following the recommended ENCODE eCLIP pipeline (https://www.encodeproject.org/pipelines/ENCPL357ADL/). For the adaptor removal of eiCLIP sequencing samples we also used ‘cutadapt’ tool (https://cutadapt.readthedocs.io/en/stable/) with the following parameters: ‘cutadapt -f fastq–match-read-wildcards–times 1 -e 0.1 -O 1–quality-cut-off 6 -m 18 -a AGATCGGAAG $INPUT.fastq>$OUTPUT.adapterTrim.fastq 2 > $OUTPUT.adapterTrim.metrics’.

Both eCLIP and eiCLIP samples were aligned by STAR alignment tool (v.2.4.2a) (https://github.com/alexdobin/STAR) with the following parameters: ‘STAR –runThreadN 8 –runMode alignReads –genomeDir GRCh38 Gencode v25 –genomeLoad LoadAndKeep –readFilesIn read1, read2, –readFilesCommand zcat –outSAMunmapped Within –outFilterMultimapNmax 1 –outFilterMultimapScoreRange 1 –outSAMattributes All –outSAMtype BAM Unsorted –outFilterType BySJout –outFilterScoreMin 10 –alignEndsType EndToEnd –outFileNamePrefix outfile’.

For the overamplification correction of eCLIP samples we used a barcode collapse python script ‘barcode_collapse_pe.py’ available on GitHub (https://github.com/YeoLab/gscripts/releases/tag/1.0). For the eiCLIP samples we used a custom python script to swap random barcodes from the first 7 nucleotides of the FASTQ sequence line to the FASTQ header line. Uniquely mapped reads with the same genomic positions and the same random barcode were then removed as PCR duplicates.

To identify binding clusters, we used cDNA-starts as cross-linking positions and as the input for False Discovery Rate clustering tool available from iMaps (https://imaps.goodwright.com/). The clusters were identified by using default parameters and Paraclu clustering algorithm (https://zenbu-wiki.gsc.riken.jp/zenbu/wiki/index.php/Paraclu). Semantic space for RBFOX2 eiCLIP targets was visualized with REVIGO^82^.

### Motif enrichment relative to eCLIP and eiCLIP crosslink sites

To identify the enrichment of RBFOX2 binding motifs relative to cross-linking sites we used density plot of already known (U)GCAUG binding motif^22,83^ relative to eiCLIP cDNA-starts from mouse liver samples and eCLIP cDNA-starts of HepG2 samples from ENCODE. Each position on the graph was normalized by the total number of mapped cDNAs from all three replicates.

### Comparison of human and mouse RBFOX2 binding sites

For the lift over of RBFOX2 crosslink clusters from mouse (mm10) to human (hg38), we used UCSC online tools (https://genome.ucsc.edu/cgi-bin/hgLiftOver). Overlap analysis between binding sites was performed with pybedtools^84,85^.

### Enrichment analysis of orthologous RBFOX2 target genes

The human orthologous genes of the mouse RBFOX2 targets detected by eiCLIP were retrieved from BioMart^86^ and investigated for enrichment for genes associated with common traits/diseases using EnrichR^87,88^.

### RNA-maps around RBFOX2-regulated exons

Alternatively spliced and control exons were selected from L^WT^ and L^Δ*Rbfox2*^ liver RNA-seq samples analysed by ‘junctionSeq’ Bioconductor package (https://bioc.ism.ac.jp/packages/3.4/bioc/html/JunctionSeq.html) from the Splicing Events table MATS.SE with the following parameters:

- Up-regulated exons: *P* < 0.05, FDR < 0.1, IncLevelDifference >0.2
- Down-regulated exons: *P* < 0.05, FDR < 0.1, IncLevelDifference <−0.2
- Control exons: *P* < 0.05, FDR < 0.1, absolute(IncLevelDifference) <0.1

For the splicing regulation analysis of RBFOX2 eiCLIP, we used an RNA-maps approach as previously published^89^. Density graphs were plotted as distribution of cDNA-starts of RBFOX2 eiCLIP samples relative to 5′ and 3′ splice sites. All three replicates were grouped together and each group of exons was normalized by the total number of exons per group.

### Statistical analysis

Differences between dietary groups and gene targets were analysed for statistical significance using a one- or two-way analysis of variance (ANOVA) test. Pairwise comparisons were analysed with two-sided Student’s *t*-test when applicable. Results are represented as mean ± s.e.m.

### Reporting summary

Further information on research design is available in the Nature Portfolio Reporting Summary linked to this article.

### Supplementary information

Reporting Summary.

Supplementary Table 1 Supplementary Tables 1–6.

### Source data

Source Data Fig. 2 Original western blot images included in Fig. 2b,c

Source Data Fig. 2 Statistical source data.

Source Data Fig. 3 Statistical source data.

Source Data Fig. 4 Statistical source data.

Source Data Fig. 5 Original western blot images included in Fig. 5a,d.

Source Data Fig. 5 Statistical Source Data

Source Data Fig. 6 Statistical source data.

Source Data Fig. 7 Statistical source data.

Source Data Fig. 7 FACS gating strategy. **a**, Live cells were gated by plotting forward scatter against side scatter. **b**, Doublets were excluded by plotting forward scatter area against forward scatter height. **c**, GFP was excited by a 488 nm laser and emission collected using 530/30 nm band pass filter. DIL was excited by a 561 nm laser and emission collected using 610/20 nm band pass filter. Samples were analysed in a BD facs aria III cytometer using BD FACSDiva 9.0.1 software.

Source Data Extended Data Fig. 2 Statistical source data.

Source Data Extended Data Fig. 3 Statistical source data.

Source Data Extended Data Fig. 4 Statistical source data.

Source Data Extended Data Fig. 5 Statistical source data.

Source Data Extended Data Fig. 5 Original western blot images included in Extended Data Fig. 5d

Source Data Extended Data Fig. 6 Statistical source data.

Source Data Extended Data Fig. 6 Original western blot images included in Extended Data Fig. 6g

Source Data Extended Data Fig. 7 Statistical source data.

Source Data Extended Data Fig. 8 Statistical source data.

Source Data Extended Data Fig. 8 Original western blot images included in Extended Data Fig. 8c.

Source Data Extended Data Fig. 9 Statistical source data.

Source Data Extended Data Fig. 9 Original western blot images included in Extended Data Fig. 9j

Source Data Extended Data Fig. 10 Statistical source data.

Source Data Extended Data Fig. 10 Original western blot images included in Extended Data Fig. 10d,h

Source Data Extended Data Fig. 10 FACS gating strategy. **a**, Live cells were gated by plotting forward scatter against side scatter. **b**, Doublets were excluded by plotting forward scatter area against forward scatter height. **c**, GFP was excited by a 488 nm laser and emission collected using 530/30 nm band pass filter. DIL was excited by a 561 nm laser and emission collected using 610/20 nm band pass filter. Samples were analysed in a BD facs aria III cytometer using BD FACSDiva 9.0.1 software.

## Data Availability

The data that support the findings on this study are available online. The mass spectrometry data have been deposited to the ProteomeXchange Consortium via the PRIDE partner repository^90^ with the dataset identifier PXD036976. RNA-seq data and eiCLIP data generated for this study have been deposited at Gene Expression Omnibus under accession number GSE151753. Source data are provided with this paper.

## Code availability

The code corresponding to Figs. 1e–g and 3b,c is publicly available at the GitHub repository (https://github.com/nebo56/RBFOX2-data_analysis).
