# Supplementary material for: Liver RBFOX2 regulates cholesterol homeostasis via Scarb1 alternative splicing in mice
Source: Nat Metab. 2022 Dec 19;4(12):1812–29. doi: 10.1038/s42255-022-00681-y (PMC9771820; doi:10.1038/s42255-022-00681-y)
Supplement: Supplementary file 1 — Reporting Summary. [file 42255_2022_681_MOESM1_ESM.pdf]

nature portfolio

Corresponding author(s): Santiago Vernia

Last updated by author(s): Sep 27, 2022

Reporting Summary

Nature Portfolio wishes to improve the reproducibility of the work that we publish. This form provides structure for consistency and transparency in reporting. For further information on Nature Portfolio policies, see our Editorial Policies and the Editorial Policy Checklist.

Statistics

For all statistical analyses, confirm that the following items are present in the figure legend, table legend, main text, or Methods section.

n/a	Confirmed	
☐	☒	The exact sample size (n) for each experimental group/condition, given as a discrete number and unit of measurement
☐	☒	A statement on whether measurements were taken from distinct samples or whether the same sample was measured repeatedly
☐	☒	The statistical test(s) used AND whether they are one- or two-sided<br>*Only common tests should be described solely by name; describe more complex techniques in the Methods section.*
☐	☒	A description of all covariates tested
☐	☒	A description of any assumptions or corrections, such as tests of normality and adjustment for multiple comparisons
☐	☒	A full description of the statistical parameters including central tendency (e.g. means) or other basic estimates (e.g. regression coefficient) AND variation (e.g. standard deviation) or associated estimates of uncertainty (e.g. confidence intervals)
☐	☒	For null hypothesis testing, the test statistic (e.g. F, t, r) with confidence intervals, effect sizes, degrees of freedom and P value noted<br>*Give P values as exact values whenever suitable.*
☒	☐	For Bayesian analysis, information on the choice of priors and Markov chain Monte Carlo settings
☒	☐	For hierarchical and complex designs, identification of the appropriate level for tests and full reporting of outcomes
☒	☐	Estimates of effect sizes (e.g. Cohen's d, Pearson's r), indicating how they were calculated

Our web collection on statistics for biologists contains articles on many of the points above.

Software and code

Policy information about availability of computer code

Data collection	-RNAseq data was processed using RTA version 1.18.54, with default filter and quality settings. The reads were demultiplexed with CASAVA 2.17. Additional information is detailed in methods section. Tophat2 (2.0.11) was used for alignement. Lipidomics data acquisition was performed with Xcalibur software (version 4.1)
Data analysis	-R 3.6.1 was applied for the RNAseq analysis. rMATS 3.2.5 was applied for the pre-mRNA alternative splicing analysis. See methods section for additional details. The code corresponding to Fig. 1E-G and Fig. 3B,C is publicly available at the GitHub repository (https://github.com/nebo56/RBFOX2-data_analysis). Both eCLIP and eiCLIP samples were aligned by STAR alignment tool (version 2.4.2a).

For manuscripts utilizing custom algorithms or software that are central to the research but not yet described in published literature, software must be made available to editors and reviewers. We strongly encourage code deposition in a community repository (e.g. GitHub). See the Nature Portfolio guidelines for submitting code & software for further information.

Data

Policy information about availability of data

All manuscripts must include a data availability statement. This statement should provide the following information, where applicable:

- Accession codes, unique identifiers, or web links for publicly available datasets
- A description of any restrictions on data availability
- For clinical datasets or third party data, please ensure that the statement adheres to our policy

The data that support the findings are publicly available: RNA-seq (Fig. 1A-B; 1D; Ext. Fig. 1B; Ext. Fig. 1D-G and Ext. Fig. 3E) and eiCLIP (Fig. 3A-I, Ext. Fig. 3A-F) data

generated for this study have been deposited at GEO under accession number GSE151753. The mass spectrometry data (Fig. 1B-C; 2D; Ext. Fig. 1A; Ext. Fig. 1C) have been deposited to the ProteomeXchange Consortium via the PRIDE partner repository with the dataset identifier PXD036976. In addition, the full list of proteins identified across the experiments has been included in Supplementary Table 1.

Human research participants

Policy information about studies involving human research participants and Sex and Gender in Research.

Reporting on sex and gender	Not applicable
Population characteristics	Not applicable
Recruitment	Not applicable
Ethics oversight	Not applicable

Note that full information on the approval of the study protocol must also be provided in the manuscript.

Field-specific reporting

Please select the one below that is the best fit for your research. If you are not sure, read the appropriate sections before making your selection.

- [x] Life sciences
- [ ] Behavioural & social sciences
- [ ] Ecological, evolutionary & environmental sciences

For a reference copy of the document with all sections, see nature.com/documents/nr-reporting-summary-flat.pdf

Life sciences study design

All studies must disclose on these points even when the disclosure is negative.

Sample size	No statistical method was applied to determine the sample size. Sample size used was based on our previous studies (e.g PMID: 35293570, PMID: 26947074 , PMID: 26910012, PMID: 25043817). The number of animals or independent experiments are indicated in figure legends.
Data exclusions	No data were excluded, unless there is a failure in measurement.
Replication	For mouse experiments, 7 to 10 samples per group were examined in one cohort, and experiments were replicated in an independent cohort. For the AAV-RBFOX2 DN and SSO in vivo treatments one cohort with the indicated number of mice was examined. RNAseq experiments were performed in a minimum of 4 samples per group. High-throughput proteomic (TMT/MS) analysis was performed in a minimum of 3 samples per group. Metabolomic analyses were performed in 5 to 6 samples per group. The other cell culture in vitro studies were performed in a minimum of 6 samples per group and experiments were replicated at least 3 times. In all data presented replication was successful.
Randomization	Mice were randomly assembled into groups determined by sex, genotype or physiological state (for example starved). For in vitro studies, when possible, samples were randomly distributed across culture plates and randomly processed to prevent potential artifacts such as batch or edge effects.
Blinding	Blinding was performed for a subset of experiments

Reporting for specific materials, systems and methods

We require information from authors about some types of materials, experimental systems and methods used in many studies. Here, indicate whether each material, system or method listed is relevant to your study. If you are not sure if a list item applies to your research, read the appropriate section before selecting a response.

Materials & experimental systems

n/a	Involved in the study	
[ ]	[x]	Antibodies
[ ]	[x]	Eukaryotic cell lines
[x]	[ ]	Palaeontology and archaeology
[ ]	[x]	Animals and other organisms
[x]	[ ]	Clinical data
[x]	[ ]	Dual use research of concern

Methods

n/a	Involved in the study	
[x]	[ ]	ChIP-seq
[x]	[ ]	Flow cytometry
[x]	[ ]	MRI-based neuroimaging

Antibodies

Antibodies used	The following antibodies were used in this study:<br>1.- For western-blot<br>- Anti-RBFOX2 antibody; Bethyl Laboratories; A300-864A, lot #2. Dil 1:1000.<br>- Anti-Tubulin antibody; Santa cruz; sc-5286, lot #k0215. Dil 1:1000.<br>- Anti-SCARB1 antibody; Abcam (EP1556Y) and Invitrogen (PA3-16805). Dil 1:1000.<br>- Anti-SREBP1 antibody; Pharmigen; 557036. Dil 1:1000.<br>- Anti-vinculin antibody; Sigma; V9131. Dil 1:1000.<br>- Anti-ACC (C83B10) antibody; Cell signaling; 3676. Dil 1:1000.<br>- Anti-pS79 ACC (D7D11) antibody; Cell signaling; 11818. Dil 1:1000.<br>- Anti-FASN (C20G5) antibody; Cell signaling; 3180. Dil 1:1000.<br>- Anti-ABCA1 antibody; Invitrogen PA116789. Dil 1:1000.<br>- Anti-APOB antibody; Proteintech 20578-1-AP. Dil 1:1000.<br>- Anti-PLA2G6 antibody; Santa cruz; sc-166616, lot #1411. Dil 1:1000.<br><br>2.-For IHF<br>- Anti-MRC1. Abcam; ab64693, lt #GR3266265-1. Dil 1:100.
Validation	All the antibodies used in this study have been obtained from commercial sources. Validation of these antibodies for each application was performed in previous publications as indicated by the manufacturer:<br>- Anti-RBFOX2 antibody; Bethyl Laboratories; A300-864A. Validated for Human and Mouse for WB and iCLIP. doi: 10.1016/j.cell.2019.06.001; doi: 10.1038/s41467-018-04559-0<br>- Anti-Tubulin antibody; Santa cruz; sc-5286. Validated for Human and Mouse for WB. doi: 10.1016/j.molcel.2021.01.004<br>- Anti-SCARB1 antibody; Abcam (EP1556Y); Validated for WB. DOI: 10.7554/eLife.52551 and Invitrogen (PA3-16805). Validated for WB (https://www.thermofisher.com/order/genome-database/dataSheetPdf?producttype=antibody&productsubtype=antibody_primary&productId=PA3-16805&version=251)<br>- Anti-SREBP1 antibody; Pharmigen; 557036. Validated for WB. DOI: 10.1016/j.cell.2020.05.053<br>- Anti-vinculin antibody; Sigma; V9131. Validated for WB. DOI: 10.1038/s41467-022-33246-4<br>- Anti-ACC (C83B10) antibody; Cell signaling; 3676. Validated for WB. DOI: 10.1038/nm.3372<br>- Anti-pS79 ACC (D7D11) antibody; Cell signaling; 11818. Validated for WB. DOI: 10.1002/dmrr.997<br>- Anti-FASN (C20G5) antibody; Cell signaling; 3180. Validated for WB. DOI: 10.1016/j.humpath.2005.11.022<br>- Anti-ABCA1 antibody; Invitrogen PA116789. Validated for WB. DOI: 10.1038/s41419-021-03544-8<br>- Anti-APOB antibody; Proteintech 20578-1-AP. Validated for WB. DOI: 10.5009/gnl19115<br>- Anti-PLA2G6 antibody; Santa cruz; sc-166616. Validated for WB. doi: 10.1007/s10620-015-3807-5; doi: 10.1194/jlr.RA119000281.<br>- Anti-MRC1. Abcam; ab64693. Validated for Mouse IF applications. doi: 10.1016/j.ccell.2019.12.003

Eukaryotic cell lines

Policy information about cell lines and Sex and Gender in Research

Cell line source(s)	Hepa 1-6 [Hepa1-6] (ATCC® CRL-1830); AML12 (ATCC® CRL-2254); Hek293 (ATCC® CRL-1573), CGT-RCiB-10 (Cell & Gene Therapy Catapult, London, U.K.)
Authentication	Cell lines were obtained from the providers and were authenticated by the providers. No further authentication was performed.
Mycoplasma contamination	Absence of mycoplasma is tested in a monthly basis.
Commonly misidentified lines<br>(See ICLAC register)	No commonly misidentified lines were used in this study.

Animals and other research organisms

Policy information about studies involving animals; ARRIVE guidelines recommended for reporting animal research, and Sex and Gender in Research

Laboratory animals	C57BL6/J (stock number 000664), Rbfox2loxP/loxP (stock number 014090) and Albumin-cre (stock number 003574) were obtained from the Jackson Laboratory (https://www.jax.org). 8-week old male and female mice were used. Mice were housed in pathogen-free barrier facilities under a 12 hour light/dark cycle at 22ºC with controlled humidity and free access to food and water. See methods for additional information.
Wild animals	The study did not involve wild animals
Reporting on sex	Sex was specified in each experiment
Field-collected samples	The study did not involve samples collected in the field

Ethics oversight	All in vivo work was approved by the animal welfare and ethical review board at Imperial College London and in accordance with the United Kingdom Animals (Scientific Procedures) Act (1986).

Note that full information on the approval of the study protocol must also be provided in the manuscript.
