# Supplementary figures and images for: Liver RBFOX2 regulates cholesterol homeostasis via Scarb1 alternative splicing in mice
Source: Nat Metab. 2022 Dec 19;4(12):1812–29. doi: 10.1038/s42255-022-00681-y (PMC9771820; doi:10.1038/s42255-022-00681-y)

**Figure 2B**

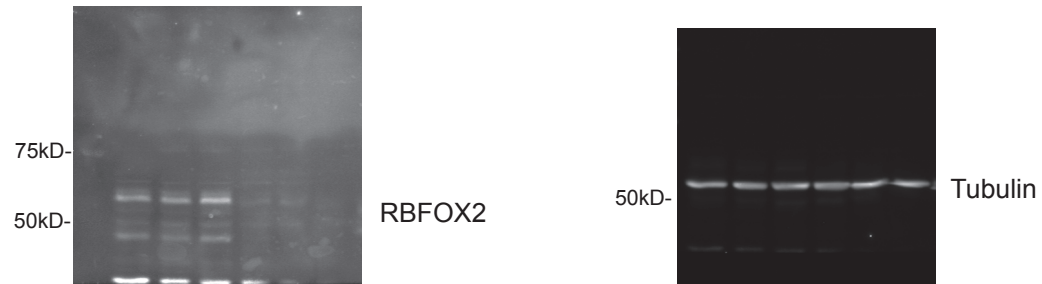

**Figure 2C**

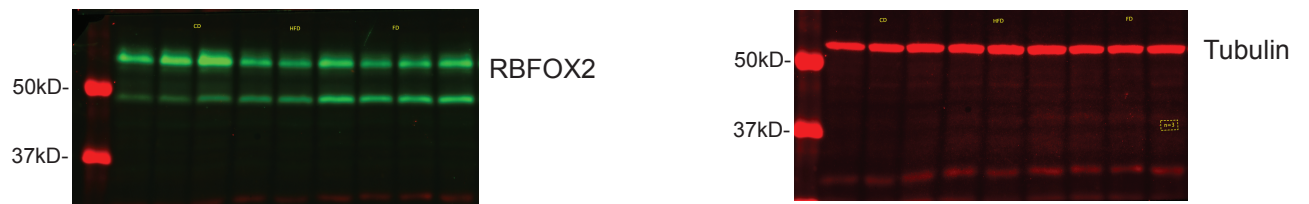

Supplement: Supplementary file 3 — Original western blot images included in Fig. 2b,c [file 42255_2022_681_MOESM3_ESM.pdf]

**Figure 5A**

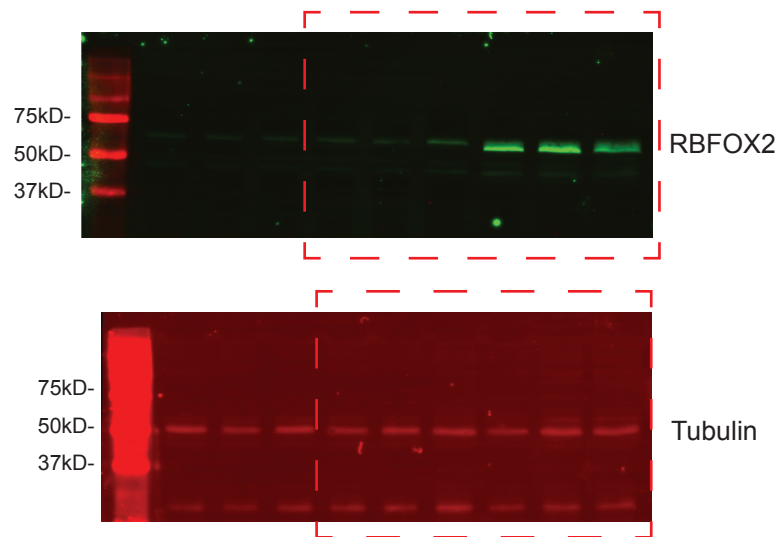

**Figure 5D**

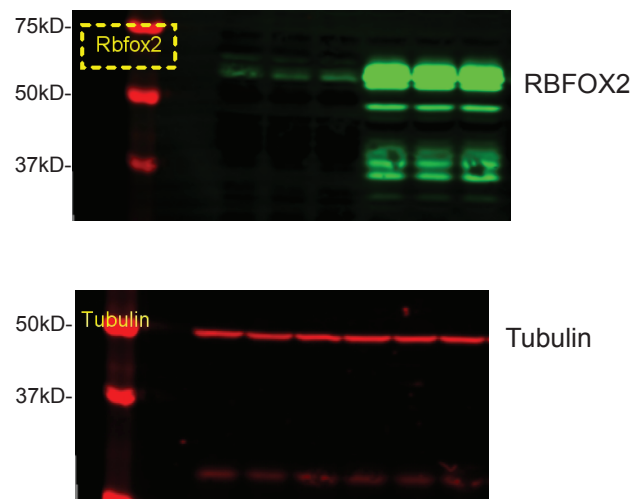

Supplement: Supplementary file 7 — Original western blot images included in Fig. 5a,d. [file 42255_2022_681_MOESM7_ESM.pdf]

Ext. Fig. 5D

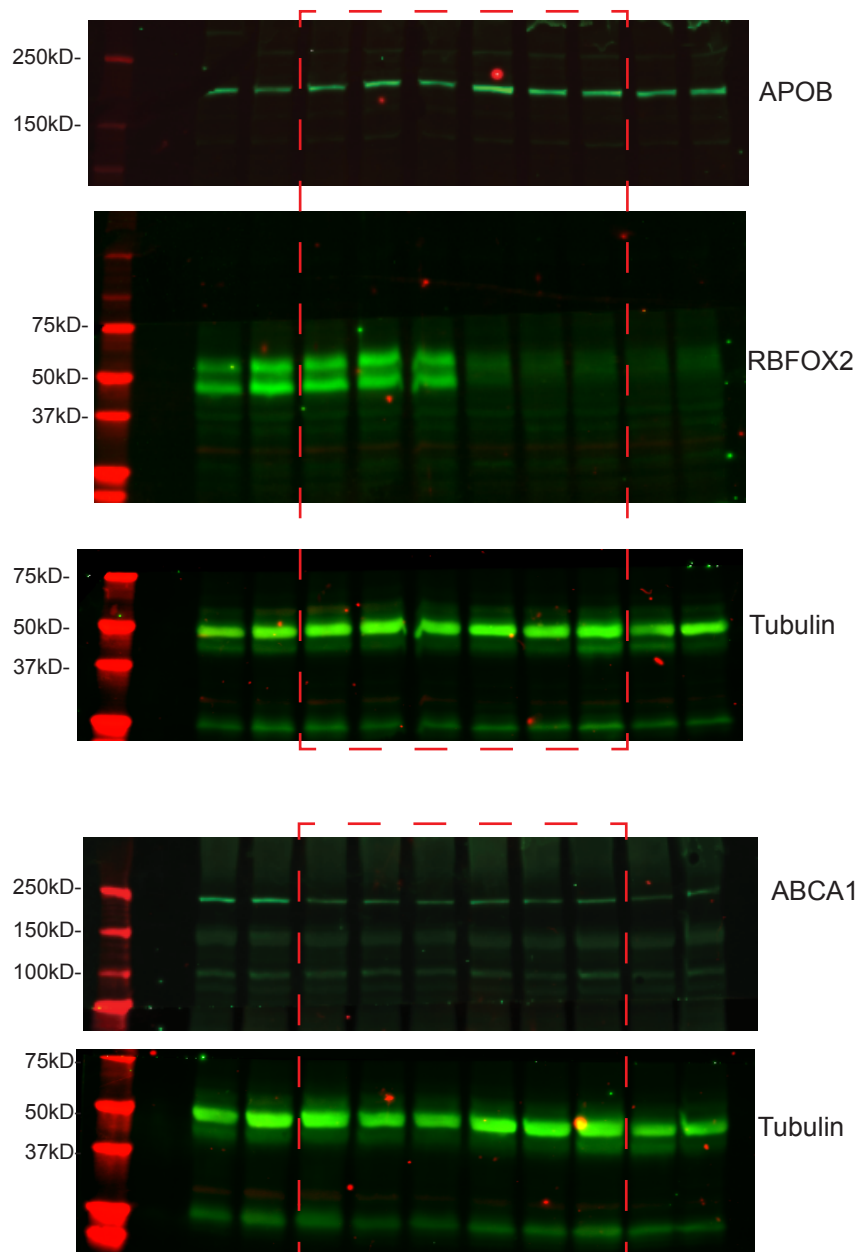

Supplement: Supplementary file 16 — Original western blot images included in Extended Data Fig. 5d [file 42255_2022_681_MOESM16_ESM.pdf]

**Ext. Fig. 6G**

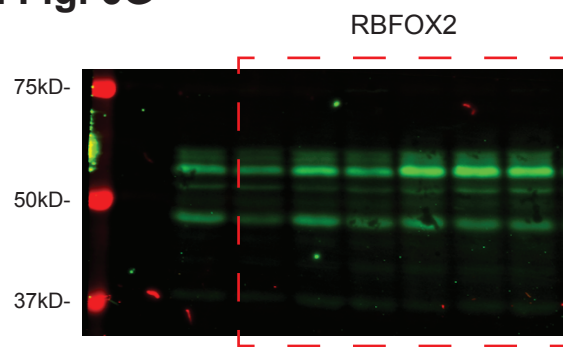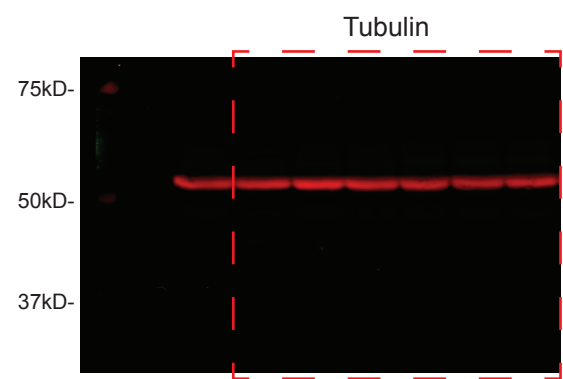

Supplement: Supplementary file 18 — Original western blot images included in Extended Data Fig. 6g [file 42255_2022_681_MOESM18_ESM.pdf]

Ext. Fig. 8C

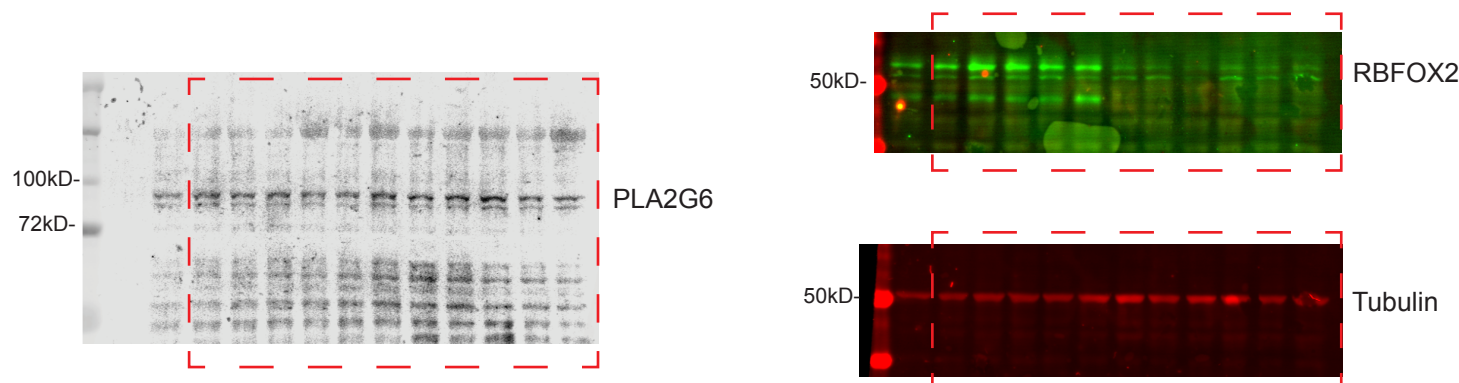

Supplement: Supplementary file 21 — Original western blot images included in Extended Data Fig. 8c. [file 42255_2022_681_MOESM21_ESM.pdf]

Ext. Fig. 9J

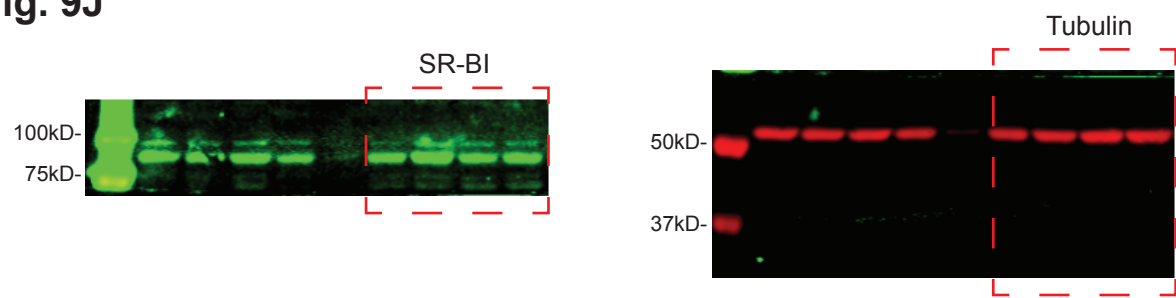

Supplement: Supplementary file 23 — Original western blot images included in Extended Data Fig. 9j [file 42255_2022_681_MOESM23_ESM.pdf]

Ext. Fig. 10D

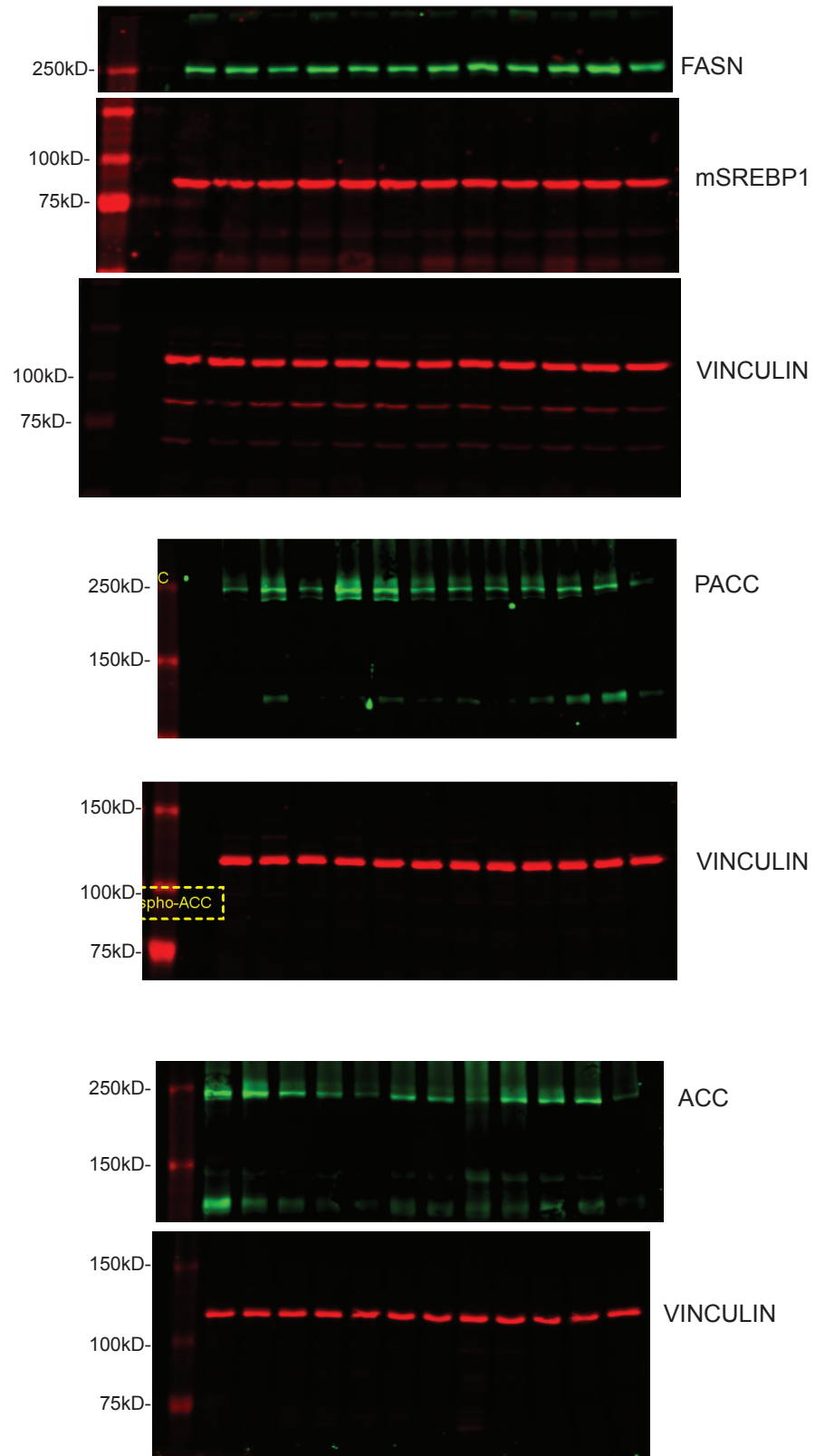

Ext. Fig. 10H

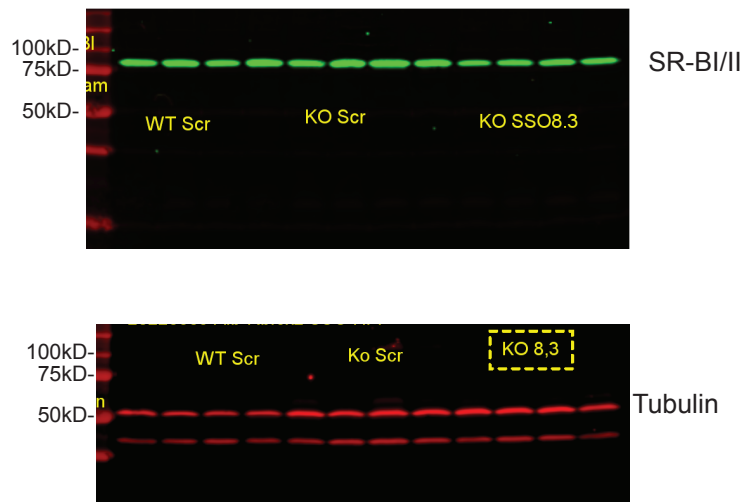

Supplement: Supplementary file 25 — Original western blot images included in Extended Data Fig. 10d,h [file 42255_2022_681_MOESM25_ESM.pdf]
