# Supplementary material for: Liver RBFOX2 regulates cholesterol homeostasis via Scarb1 alternative splicing in mice
Source: Nat Metab. 2022 Dec 19;4(12):1812–29. doi: 10.1038/s42255-022-00681-y (PMC9771820; doi:10.1038/s42255-022-00681-y)
Supplement: Supplementary file 11 — FACS gating strategy. a, Live cells were gated by plotting forward scatter against side scatter. b, Doublets were excluded by plotting forward scatter area against forward scatter height. c, GFP was excited by a 488 nm laser and emission collected using 530/30 nm band pass filter. DIL was excited by a 561 nm laser and emission collected using 610/20 nm band pass filter. Samples were analysed in a BD facs aria III cytometer using BD FACSDiva 9.0.1 software. [file 42255_2022_681_MOESM11_ESM.pdf]

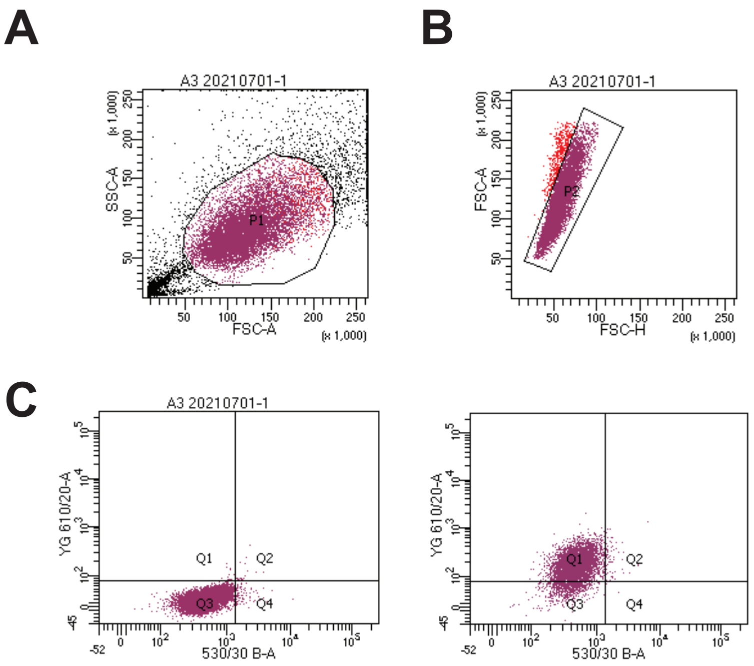

A.-Live cells were gated by plotting forward scatter against side scatter. B.- Doublets were excluded by plotting forward scatter area against forward scatter height. C.- GFP was excited by a 488nm laser and emission collected using 530/30nm band pass filter. DIL was excited by a 561nm laser and emission collected using 610/20nm band pass filter. Samples were analysed in a BD facs aria III cytometer using BD FACSDiva 9.0.1 software.
